# Spinal cord tissueoid transplantation combined with tail nerve electrical stimulation promotes the voluntary movement of paralyzed hindlimbs in rats with transected spinal cord injury

**DOI:** 10.1016/j.mtbio.2026.103418

**Published:** 2026-06-29

**Authors:** Bi-Qin Lai, Chuang-Ran Wu, Shang-Bin Yang, Jing Xu, Yue Yang, Rong-Jie Wu, Hai-Yang Yu, Zhen Chen, Rui Liu, Ying Ding, Ge Li, Xiang Zeng, Yuan-Huan Ma, Shan-Shan Ma, Qiao-Ying Huang, Ya-Qiong Wang, Ling Zhang, Zheng-Hong Chen, Yi-Nan Guo, Yuan-Feng Chen, Jia-Feng Fang, Qiu-Jian Zheng, Yuan-Shan Zeng

**Affiliations:** a Center for Stem Cell Biology and Tissue Engineering, Key Laboratory for Stem Cells and Tissue Engineering Ministry of Education, Department of Histoembryology and Cell Biology, Zhongshan School of Medicine, Sun Yat-sen University, Guangzhou, China; b Department of Orthopedics, Guangdong Provincial People's Hospital (Guangdong Academy of Medical Sciences), Southern Medical University, Guangzhou, 510100, China; c Department of Gastrointestinal Surgery, The Third Affiliated Hospital, Sun Yat-sen University, Guangzhou, 510630, China; d Co-innovation Center of Neuroregeneration, Nantong University, Nantong, 226001, China; e Guangdong Provincial Key Laboratory of Brain Function and Disease, Zhongshan School of Medicine, Sun Yat-sen University, Guangzhou, 510080, China; f Medical Research Institute, Guangdong Provincial People's Hospital (Guangdong Academy of Medical Sciences), Southern Medical University, Guangzhou, 510100, China; g Department of Geriatrics, Rehabilitation Medicine Department, The First Affiliated Hospital, Sun Yat-sen University, Guangzhou, 510080, China

**Keywords:** Spinal cord tissueoid, Tail nerve electrical stimulation, Transected spinal cord injury, Voluntary motor function recovery, Central pattern generator, Sensorimotor neural circuit

## Abstract

The recovery of voluntary movement after complete spinal cord injury (SCI) remains a formidable clinical challenge, as it necessitates both the reconstruction of disrupted spinal cord neural pathways and the restoration of the excitatory/inhibitory balance in sensorimotor neural circuits. To tackle this dual challenge, we transplanted a biomimetic spinal cord tissueoid (SCToid) into the injury cavity to structurally reestablish neural pathways, while concurrently applying tail nerve electrical stimulation (TNES) to functionally reactivate silent sensorimotor neural circuits. The results showed that combined SCToid transplantation and TNES promoted the regeneration of corticospinal tract and sensory afferent axons, which formed functional synapses with SCToid neurons. Moreover, monosynaptic tracer assays revealed direct innervation of lumbar spinal cord central pattern generator (CPG) interneurons by SCToid neurons; some CPG interneurons and sensory afferent axons also exhibited synaptic connectivity with motor neurons. Compared with the control group, transplantation of SCToids combined with TNES increased the ratio of excitatory/inhibitory synaptic terminals on the soma surfaces of CPG interneurons and motor neurons toward the pattern observed in normal spinal cords. This change ultimately enhanced the excitability of sensorimotor neural circuits and restored weight-bearing hindlimb walking. Collectively, these findings establish that reconstructing neural pathways and restoring the excitatory/inhibitory balance within CPG-regulated sensorimotor neural circuits are both necessary and sufficient to enable voluntary movement recovery. This synergistic mechanism establishes a robust theoretical framework for integrating biological and physical therapeutic strategies in SCI treatment, with specific implications for the combined application of transplantable engineered organoids and neurostimulation-based rehabilitation approaches.

## Graphical abstract

**Figure:**
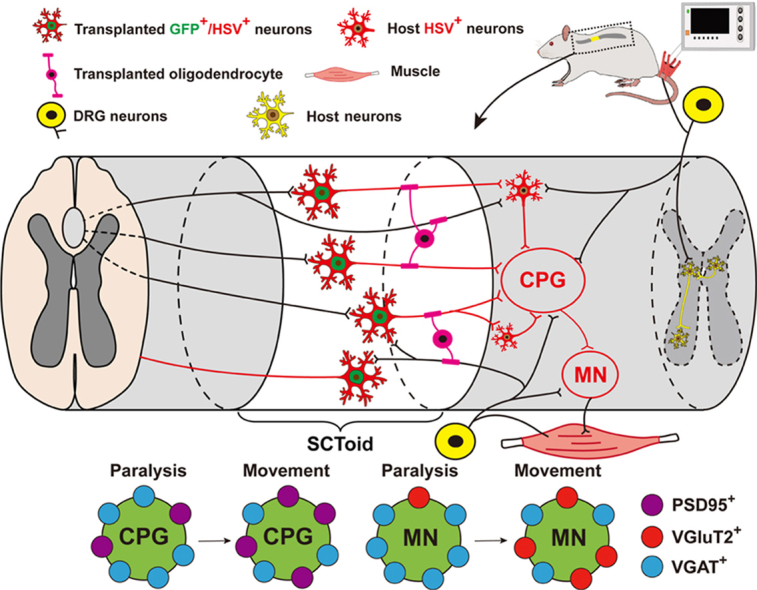

## 1 Introduction

Spinal cord transection results in complete disruption of ascending and descending neural pathways and loss of brain communication with the spinal cord caudal to the lesion [1,2]. Spinal cord contusion models better mimic clinical injury but cannot distinguish whether functional recovery arises from axon regeneration or preservation of residual pathways; spinal cord transection models, by contrast, are ideal for studying functional reconstruction driven by axon regeneration and information relay [3,4]. In studies investigating spinal cord transection, although various stem cell-based tissue engineering strategies have been developed [[5], [6], [7], [8]], achieving structural and functional reconstruction of the injured spinal cord remains challenging.

In recent years, spinal cord organoids, spinal cord-like tissue (SCLT), and three-dimensional-printed spinal cord tissue have been used for transplantation to reconstruct neural pathways [5,8,9]. Transplanted neurons have been showed to survive and form synaptic connections with regenerating axons [10,11]. However, paralyzed limbs exhibited only slight movement, and weight-bearing function and recovery of voluntary movement remained difficult. One reason is poor functional integration of xenogeneic or allogeneic transplanted neurons with the host spinal cord [[12], [13], [14]]. Another reason is functional silencing of sensorimotor neural circuits and skeletal muscle atrophy in paralyzed limbs [5,15,16].

Recent studies have showed that for some patients with thoracic SCI, even without neural pathway repair, assisted walking can be achieved under programmed epidural electrical stimulation (EES) of the lumbar spinal cord [[17], [18], [19]]. These studies also suggested that the key to movement achieved by EES is the activation of central pattern generator (CPG)-regulated sensorimotor neural circuits [20,21]. However, EES fails to achieve optimal outcomes in patients with lumbar spinal cord neurodegeneration and severe lower limb muscle atrophy. In such circumstances, nerve electrical stimulation combined with neural pathway reconstruction is necessary [[22], [23], [24]].

Our previous study showed that SCLT transplantation combined with tail nerve electrical stimulation (TNES) activates sensorimotor neural circuits in the lumbar spinal cord, effectively preventing skeletal muscle atrophy and significantly improving locomotor function in rats [24]. The SCLT is constructed using a collagen sponge (CS) scaffold, which exhibits softness and an isotropic porous architecture with a pore size range of 50–100 μm. The non-oriented channels within the CS scaffold facilitate the uniform three-dimensional distribution of neural stem cells (NSCs), promote their directed differentiation into functional neurons, and support the development of extensive, dense neurite branches as well as localized synaptic networks—thereby more faithfully recapitulating the spinal cord gray matter. However, the white matter-like tissue of the SCLT, which is constructed based on the CS scaffold, cannot provide longitudinal channels for regenerating axons [8,24]. Our recent research has found that a natural bioactive material (decellularized optic nerve, DON) can be used as a scaffold for tissue-engineered spinal white matter-like tissue (WMLT) and features uniformly distributed longitudinal channels, making it particularly suitable for straight growth of regenerated axons [25]. In addition, how TNES, which shares functional similarities with EES, synergistically promotes synaptic formation between transplanted neurons and CPG-regulated sensorimotor neural circuits remains unclear [26,27].

The optic nerve is a cranial nerve composed primarily of longitudinally aligned retinal ganglion cell axons, which are ensheathed by oligodendrocyte-derived myelin sheaths [28]. In the present study, we used a laminin (LN)- and type IV collagen (Col IV)-rich DON scaffold, which mimics the extracellular matrix (ECM) and axon tract growth channels of developing optic nerves [29], to prepare spinal cord white matter–like tissue and a CS scaffold to prepare spinal cord gray matter–like tissue. NSCs were subsequently seeded into both scaffolds to construct a spinal cord tissueoid (SCToid) *in vitro*. Tissueoids are three-dimensional tissue-mimetic *in vitro* models engineered using stem cells or tissue engineering techniques, with defined architecture and function. Unlike organoids, which recapitulate multi-lineage integration and systemic physiology of natural organs, tissueoids emphasize tissue-scale functional reconstruction and biological fidelity [30]. We hypothesized that SCToid transplantation would replace damaged spinal cord tissue and reconstruct interrupted neural pathways, while TNES would enhance synaptic connectivity between SCToid neurons and sensorimotor neural circuits to facilitate voluntary locomotor recovery.

## 2 Materials and methods

### 2.1 Culture and identification of NSCs

All animal experiments were conducted in accordance with the guidelines for the Animal Care and Use Committee of Sun Yat-sen University (SYSU-IACUC-2022-B1104). Neural stem cells (NSCs) were procured from either Sprague-Dawley (SD) rats or green fluorescent protein-positive (GFP) transgenic SD rats, provideed by Osaka University (Osaka, Japan), following previously established protocols [31]. Briefly, rats at postnatal day 1 were anesthetized, and hippocampal tissue was dissected and mechanically triturated to obtain a single-cell suspension. Cells were cultured in DMEM/F12 (1:1) supplemented with 1% B27 (Life Technologies, Carlsbad, CA, USA) and 20 ng/mL basic fibroblast growth factor (bFGF, Life Technologies, Gaithersburg, MD, USA). This medium is selective and eliminates adherent cells and serum-dependent cells. Cells proliferated in suspension, forming neurospheres. After 5 days, all neurospheres were subjected to Nestin immunoreactivity testing.

### 2.2 Culture and identification of OPCs

Neural stem cells (NSCs) were induced into oligodendrocyte progenitor cells (OPCs) using the established protocol [8,32]. Briefly, Nestin-positive neurospheres were cultured with DMEM/F12 containing 10 ng/mL platelet-derived growth factor AA (PDGF-AA, PeproTech, NJ, USA), 10 ng/mL bFGF, 30 ng/mL triiodothyronine (T3, Sigma-Aldrich, MO, USA), and 1% fetal bovine serum (FBS, Gibco, NY, USA). After 3 days, OPCs were purified by differential digestion-adhesion, exploiting their higher trypsin sensitivity and delayed re-adhesion compared with other cell types. Trypsin digestion was halted when ∼70% of cells had detached; the suspension was then transferred to 1% FBS-containing medium and incubated for 2 h to allow non-OPC contaminants to adhere. Non-adherent cells were collected and replated in fresh flasks for expansion to obtain purified OPCs. Cells were immunostained for neuron-glia antigen 2 (NG2) (EMD Millipore, MA, USA). The purity of the OPCs used in all subsequent experiments was ∼80% NG2^+^ immunoreactivity.

### 2.3 Gene transfection and overexpression

NSCs were transfected with an adeno-associated virus-DJ‌ (AAV-DJ) carrying the *neurotrophin-3 (NT-3)* coding sequence (MOI:1000, NM_002527, pAV-CMV-NT-3-P2A-GFP, Vigene Biosciences) and an AAV-DJ carrying the tyrosine kinase receptor C (TrkC) coding sequence (MOI:1500, NM_001007156, pAV-CMV-TrkC-P2A-GFP, Vigene Biosciences, Shandong, China). OPCs were genetically modified with a ciliary neurotrophic factor (CNTF) gene delivered via AAV-DJ (MOI:1000, NM_000614 pAV-CMV-CNTF-P2A-GFP, Vigene Biosciences, Shandong, China). Immunofluorescence detection of NT-3, TrkC, and CNTF was used to evaluate and identify the cells successfully transfected with AAV-DJ.

### 2.4 Preparation and identification of decellularized optic nerve (DON)

Fresh optic nerves extending from the eyeball to the optic chiasm were harvested from adult pigs (approximately 2 years old; supplied by Northwest A&F University, China). Following removal of adipose tissue and the dura mater, the nerves were cut into 2-cm segments. To remove cellular and nuclear components, a sequential detergent-enzyme decellularization protocol was performed using 3% Triton X-100 in aqueous solution for 12 h, 4% sodium deoxycholate in aqueous solution for 24 h, and a solution containing 50 U/mL DNase I (Sigma-Aldrich, MO, USA) and 10 μg/mL RNase A (Sigma-Aldrich, MO, USA) for 3 h. The tissues were then lyophilized for ≥24 h and stored at 4°C until further use. To confirm the complete removal of cellular material, Hoechst33342 (Hoe) nuclear staining was performed and assessed under fluorescence microscopy.

### 2.5 Construction of the SCToid and SCLT

The design concepts of SCToid and SCLT are rooted in the biomimetic engineering of the natural anatomical architecture of spinal cord tissue—characterized by an outer envelope of white matter and a central core of gray matter. For gray matter-like tissue (GMLT) construction, the scaffold is molded into a cylindrical structure, with its diameter precisely defined based on the measured anteroposterior dimension of the gray matter in the target rat spinal cord segment. This scaffold serves as a three-dimensional carrier for seeding genetically modified NSCs and directing their lineage-specific differentiation toward a neuronal phenotype. In contrast, white matter-like tissue (WMLT) construction involves engineering the scaffold into a concentric tubular configuration, wherein the inner diameter is precisely matched to that of the GMLT and the outer diameter is aligned with the native diameter of the host spinal cord tissue, thereby enabling seamless morphological integration. This tubular scaffold is specifically designed to accommodate genetically modified OPCs and support their directed differentiation into mature oligodendrocytes. Following completion of lineage-specific differentiation in both GMLT and WMLT, the pre-differentiated cylindrical GMLT is inserted into the inner lumen of the tubular WMLT, resulting in the assembly of SCToid or SCLT with compartmentalized functional units. The detailed construction workflow is as follows:

We constructed an SCToid composed of WMLT and gray matter-like tissue (GMLT). The SCToid was formed using DON as a white matter-like scaffold and collagen sponge (CS, BIOT Biology, Wuxi, China) as a gray matter-like scaffold. For WMLT fabrication, circular DON (3 mm outer diameter, 2 mm inner diameter, 2 mm height) was seeded with 20 μL of CNTF-OPC suspension (2 × 10^5^ cells) and cultured for 7 days in DMEM/F12 supplemented with 10 ng/mL bFGF, 10 ng/mL PDGF-AA, 30 ng/mL T3, and 1% FBS—conditions promoting OPC colonization and their differentiation into immature oligodendrocytes [8]. Concurrently, GMLT was generated by seeding pre-hydrated CS cylinders (2 mm diameter, 2 mm height) with 20 μL of NT-3/TrkC-NSC suspension (2 × 10^5^ cells) and culturing in Neural Basal Medium + 1% FBS for 7 days—supporting colonization of NT-3/TrkC-NSCs on the scaffold and their differentiation into immature neurons [8]. The parallel SCLT constructs employs CS as the scaffold material for both WMLT and GMLT, using the same type of seed cells as the SCToid.

Following a 7-day independent culture period, the cylindrical GMLT was embedded into the lumen of the tubular WMLT, yielding composite SCToid or SCLT constructs. These assembled constructs underwent an additional 7-day culture phase in DMEM/F12 supplemented with B27 and 5% FBS. The 7-day co-culture period is sufficient to promote cellular interaction, maturation, and structural-functional integration of WMLT and GMLT [8]. Finally, to elucidate the role of integrin receptor-mediated cell-ECM interactions in neuronal differentiation and development, the culture medium—which was replaced every other day—was supplemented with specific blocking antibodies designed to inhibit integrin subunit alpha v (ITG-αv antibody; 1:300; BioLegend, San Diego, CA, USA; Cat. No. 304208) and integrin subunit beta 1 (ITG-β1 antibody; 1:200; Santa Cruz Biotechnology, Dallas, TX, USA; Cat. No. sc-53711). Blocking antibodies were added at 24 h post-assembly of WMLT and GMLT.

### 2.6 Spinal cord transection and transplantation

All animal experiments were conducted in accordance with the guidelines of the Animal Care and Use Committee of Sun Yat-sen University (SYSU-IACUC-2022-B1104). We chose adult female SD rats for the complete transection SCI model, because postoperative bladder expression is technically challenging in male rats. Three days before surgery, subcutaneous injections of cyclosporin A (20 mg/kg) were administered in the abdominal region of the rats. Following anesthesia (1% sodium pentobarbital, 40 mg/kg), a laminectomy was performed at the T9 vertebral level, followed by a complete 2 mm transection at the T10 spinal cord segment. After hemostasis, the DON (without cells loaded), SCLT, and SCToid were transplanted into the injury site of the spinal cord, respectively, the rats were then assigned to the SCI, DON, SCLT, and SCToid groups. Treatment group compositions are listed in Supplementary Table 1. In the SCI group, a 2-mm complete transection was performed without transplantation of any biomaterials. Comprehensive postoperative care was administered to all rats, which included daily intramuscular injections of penicillin (80,000 units) and an analgesic (Meloxicam, 0.1 mg/kg) for one week. Manual bladder expression was performed as needed until the restoration of autonomous urination was observed. Furthermore, cyclosporin A was administered to all animals daily for two consecutive months.

### 2.7 TNES treatment

Rats were randomized into five groups—the SCI (only spinal cord injury), DON (SCI + DON transplantation), SCLT (SCI + SCLT transplantation), SCToid (SCI + SCToid transplantation), and Nor (uninjured controls) groups—with TNES treatment administered according to group-specific protocols post-surgery. Each main group included a corresponding control subgroup that received no TNES treatment. TNES treatment commenced on postoperative day 8 [24,33]. At initiation of treatment, each rat was placed in an open-field environment. The therapy was administered using a physical therapy device (Model J18A1, Shenzhen Quanrikang Medical Equipment Co., Ltd., China). Firstly, the two electrodes were placed at the tail root of the rat, maintaining a distance of more than 1 cm. Secondly, the stimulation frequency was set to 4 kHz. During stimulation, the appropriate intensity (not exceeding 20 mA) was adjusted based on the slight contraction of the hindlimb muscles. After initiation of treatment, the rats were observed for adverse reactions. Each treatment session lasted 20 min and was conducted five times per week for a total of 8 weeks (for details of the TNES procedure, see Supplementary Video 1).

### 2.8 Behavioral testing

A weekly assessment of hindlimb joint movement, walking ability, coordination, and stability in rats following SCI was conducted using a 5-min BBB open-field locomotor test [34], employing a double-blind approach. The BBB score was calculated as the average of the scores of the left and right hindlimbs. In the double-blind test, the testers only knew the identifications (IDs) of each rat and were unaware of the group allocations. During the scoring process, a 5-min video of each rat was recorded and sent to a second scorer, who also knew only the rat IDs. The statistician averaged the scores from both scorers and analyzed them by groups. Additionally, a modified inclined grid climbing test was employed at the eight-week time point to observe and capture coordinated forelimb–hindlimb movements, providing an evaluation of each rat's overall motor function [35]. For this test, each rat was placed on an inclined grid at a 45° angle. The total number of grid frames grasped by the hindlimbs during the grid climbing test was recorded. Each rat underwent three experiments of the grid climbing tests.

### 2.9 Electrophysiological analysis

Prior to perfusion, cortical motor-evoked potentials (CMEPs) were recorded to evaluate the functional integrity of motor signaling pathways, in accordance with previously described methodology [31]. In brief, following induction of general anesthesia and surgical exposure of the sensorimotor cortex (SMC) and sciatic nerve in animals from all experimental and normal (Nor) groups, the stimulating electrode was placed in the SMC and the recording electrode was interfaced with the sciatic nerve. The resultant CMEPs were captured utilizing a NeuroExam M − 800 Data Acquisition and Analysis System (MEDCOM, Zhuhai, China).

### 2.10 BDA tracing

At eight weeks post-surgery, five rats from each experimental group underwent anterograde tracing using biotinylated dextran amine (BDA). Following anesthesia, the head of each animal was secured in a stereotaxic apparatus. Guided by a stereotaxic brain atlas, two circular craniotomies (radius = 2.5 mm) were performed on both cerebral hemispheres-located 1 mm rostral to the bregma and 1 mm lateral to the midline-to expose the underlying SMC. A 10% BDA solution (MW 10,000; Invitrogen, D1956; Thermo Fisher Scientific, Waltham, MA, USA) was administered via slow microinjection. For each hemisphere, injections were performed at six sites, set at 1, 2, and 3 mm caudal to the bregma and 1 or 2 mm lateral to the midline. At each site, 0.25 μL was delivered at depths of 2 mm and 1 mm below the cortical surface, for a total volume of 0.50 μL per site. After a 2-min interval, the needle was slowly withdrawn.

### 2.11 Anterograde monosynaptic tracing

To investigate the monosynaptic connectivity of transplanted SCToid neurons with host neurons, we introduced AAV-helper virus (rAAV-Ef1α-EGFP-T2A-TK-WPRE-hGH polyA, Brain VTA, Wuhan, China) that expresses the thymidine kinase (*TK*) gene in a trans-complementary manner into the neurons seven days prior to SCToid transplantation. Following the surgical procedure described above, transmonosynaptic herpes simplex virus (HSV, H129ΔTK-ubc-tdTomato) with the *TK* gene deleted (HSV-ΔTK, Brain VTA, China) was injected into the area rostral to the injury/graft site of the spinal cord 7 days before perfusion and fixation of the experimental animals.

### 2.12 CTB tracing

Seven days prior to perfusion, the rats were anesthetized with with 1% sodium pentobarbital, administered at a dose of 40 mg/kg. A retrograde tracer, 2% cholera toxin subunit B (CTB) was then administered. A total volume of 2 μL was administered gradually into the tail via multiple subcutaneous injections, with each injection site receiving 0.1 μL. After a seven-day transport period, the animals were euthanized.

### 2.13 Skeletal muscle wet weight measurement

Animals were terminally anesthetized (1% sodium pentobarbital, 50 mg/kg i.p.) and subjected to transcardiac perfusion. After circulatory clearance, fixation was performed with 4% paraformaldehyde in PBS. Tibialis anterior and gastrocnemius muscles were dissected (tendons preserved), post-fixed overnight at 4°C, dehydrated in sucrose, and weighed.

### 2.14 Immunocytochemistry analysis

To visualize specific proteins, immunocytochemical staining was conducted in accordance with previously published methodologies [31]. A consistent protocol was applied to both *in vitro* cell preparations and *in vivo* tissue sections, beginning with fixation in 4% paraformaldehyde. Following fixation, the samples were incubated overnight at 4°C with primary antibodies diluted in 0.01 M phosphate-buffered saline (PBS) containing 0.3% Triton X-100 to ensure membrane permeabilization. This was followed by an incubation period with the corresponding secondary antibodies. For cell nucleus visualization, the Hoechst33342 counterstain was applied. The prepared slides were subsequently imaged and analyzed using a fluorescence microscopy (Ti2-E, NIKON, Japan) or a confocal laser scanning microscope (Dragonfly, Andor Technology, Belfast, UK). A comprehensive list of the antibodies employed for this analysis is available in Supplementary Table 2.

### 2.15 Western blot analysis

The quantification of protein expression was achieved by isolating proteins from the WMLT and GMLT components of the SCLT and SCToid groups *in vitro*. Protein lysates from WMLT/GMLT components (SCLT and SCToid groups) were separated by 10% sodium dodecyl sulfate-polyacrylamide gel electrophoresis (SDS-PAGE) and transferred to polyvinylidene fluoride (PVDF) membranes. After blocking, the membranes were incubated with primary antibodies overnight at 4°C, followed by HRP-conjugated secondary antibodies. Immunoreactive bands were detected using enhanced chemiluminescence (ECL), and protein levels were normalized to glyceraldehyde 3-phosphate dehydrogenase (GAPDH). A list of antibodies is provided in Supplementary Table 2.

### 2.16 mRNA sequencing

After two weeks of cultivation *in vitro*, SCLT, SCToid and normal spinal cord (Normal control) of newborn rats were harvested and subjected to mRNA sequencing.

Total RNA was extracted and subjected to quality control: agarose gel electrophoresis (28S:18S ≥ 1.5), NanoDrop (A260/A280: 1.8-2.2), and Qubit quantification (≥500 ng/μl). Qualified samples were outsourced for RNA-Seq (Illumina platform). Raw data were processed via CASAVA base calling, followed by FPKM quantification using StringTie. Differentially expressed genes were identified with DESeq2 (|log2FC| ≥ 1, *P* ≤ 0.05).

### 2.17 Ultrastructure observation

The DON and CS scaffolds were placed in a −80°C freezer for cryopreservation and transferred to a freeze dryer for 48 h of lyophilization. The dried samples were sputter-coated with gold and examined by a scanning electron microscope (SEM, Crossbeam 550, ZEISS, Germany).

Animals were initially deeply anesthetized and then transcardially perfused with heparinized 0.1 M PBS (187.5 U/100 mL) to exsanguinate the vasculature, after which a mixed fixative solution containing 4% paraformaldehyde, 15% saturated picric acid and 0.1% glutaraldehyde was applied. The spinal cord was then dissected and post-fixed overnight at 4°C in the same fresh fixative. The tissue was sectioned into 50 μm sagittal free-floating slices using a vibratome. Sections underwent cryoprotection and permeabilization via overnight incubation at 4°C in 25% sucrose and 10% glycerol in PBS, followed by three successive freeze-thaw cycles in liquid nitrogen. Non-specific binding sites were blocked by incubating the sections for 1 h in 20% normal goat serum in Tris-buffered saline (TBS, pH 7.4). The sections were then incubated for 24 h at 4°C with a cocktail of primary antibodies, specifically anti-GFP and anti-CGRP, diluted in 2% goat serum. After thorough washing, the sections were incubated with species-specific secondary antibodies overnight at 4°C. Finally, to stabilize immunocomplexes, the sections were post-fixed with 1% glutaraldehyde for 10 min.

Antigen-antibody complexes were then visualized using an SABC-DAB kit (SA1021, BOSTER, China) or GoldEnhance™ kit (GoldEnhance™ EM Plus, Nanoprobes, NY, USA). For the gold enhancement test, the reagent was prepared by mixing four components—namely solution A (enhancer), solution B (activator), solution C (initiator), and solution D (buffer)—in equal volumes. To prepare the gold enhancement reagent, 10 μL each of solution A and solution B were mixed and incubated at room temperature for 5 min. Then, 10 μL of solution C and 10 μL of solution D were added sequentially and thoroughly mixed. The resulting mixture was applied to the tissue sections and incubated at room temperature for approximately 20 min; the exact incubation time was adjusted empirically based on staining intensity. Finally, the reaction was terminated by rinsing with distilled water. Sections were then post-fixed with osmium tetroxide, dehydrated in a graded ethanol series, and embedded in Epon 812. Ultrathin sections (70 nm) were examined using a transmission electron microscope (JEM-1400, JEOL, Japan).

### 2.18 Morphological quantification

To quantify the phenotypes of positive cells *in vitro*, five random fields of view were selected from each section across the complete series of sections for each implant (*n* = 6 in each group). Following immunocytochemistry, the percentage of Map2^+^, GFAP^+^, and Olig2^+^ cells relative to GFP^+^ cells was calculated per group.

All fluorescence images used for statistical analysis of the same detection target in each group were acquired under identical imaging parameters. For quantification, the area of interest (AOI) was delineated, and the optical density of fluorescence within the AOI was measured and analyzed using ImageJ software. To quantify the relative density of positive markers *in vitro*, six fields of view were selected from each section in the entire series of sections for each implant (SCLT, SCToid, SCToid-ITG-β1-inhibit or SCToid-ITG-αv-inhibit, *n* = 5 in each group). The fluorescence density of each field was calculated as the area occupied by specific immunoreactivity divided by the total field area. Relative fluorescence density was calculated for postsynaptic density protein 95-positive (PSD95^+^) and synaptophysin-positive (SYP^+^) staining in each group using ImageJ software.

*In vivo* the quantification of neurofilament-positive (NF^+^) axons and myelin basic protein-positive (MBP^+^) sheaths was performed on horizontal spinal cord sections. For this analysis, three distinct 2-mm regions were evaluated: the injury/graft epicenter, as well as the tissue areas immediately rostral and caudal to this site. A systematic sampling method was applied, whereby one of every five consecutive sections from each animal was processed for analysis, yielding four sections per rat (*n* = 5 rats per group). Using ImageJ software, the NF^+^ and MBP^+^ immunoreactive areas were defined as AOI. The extent of axonal presence and myelination was then determined by calculating the total pixel area of each respective AOI.

To quantify the percentage of immunopositive structures *in vivo*, we selected one in every five sections from each rat (*n* = 5 per group). After immunofluorescence staining for specific markers, areas measuring 0.7 × 0.5 mm were chosen for each section, including two areas rostral or caudal to the injury/graft site and four areas in the injury/graft site. The positive areas for SYP^+^, ITG-αv^+^ and ITG-β1^+^ were calculated and divided by the total GFP^+^ area.

For the quantification of calcitonin gene-related peptide positive (CGRP^+^ axons) *in vivo*, each group consisted of 5 rats, each with 2 longitudinal sections, and the number of CGRP^+^ axons (length greater than 40 μm) was counted per visual field in the injury/graft site. For the quantification of BDA^+^ axons, tissue near the central canal of the spinal cord was collected from rats in each group. Three longitudinal sections were taken from each rat (*n* = 3 per group), and areas measuring 0.7 × 0.5 mm were taken at the areas rostral and caudal to/in the injury/graft site in each section. The number of BDA^+^ axons (length greater than 25 μm) was counted per visual field.

To investigate changes in neural plasticity within the lumbar (L) spinal cord, L2 segment was harvested from each rat, and four sections per rat were selected for statistical analysis (*n = *3 per group). For each section, a 0.7 × 0.5 mm field of view (FOV) encompassing the intermediate gray matter of the spinal cord was selected. The number of erythropoietin-producing hepatocellular carcinoma A4-and vesicular glutamate transporter 2-positive (EPHA4^+^/VGluT2^+^) double-positive neurons—a subtype of excitatory CPG interneurons—was quantified within the FOV, along with the number of PSD95^+^ or vesicular γ-aminobutyric acid transporter-positive (VGAT^+^) synaptic terminals in direct contact with the soma surface of EPHA4^+^/VGluT2^+^ neurons. The average number of PSD95^+^ or VGAT^+^ synaptic terminals per soma surface of EPHA4^+^/VGluT2^+^ neuron in the FOV was then calculated, defined as the ratio of the total number of such synaptic terminals to the total number of EPHA4^+^/VGluT2^+^ neurons. In addition, the L4 segment was harvested from each rat, and four sections were selected per rat for statistical analysis (*n = *3 per group). In each section, a 0.7 × 0.5 mm FOV encompassing the choline acetyltransferase-positive (ChAT^+^) region in the ventral horn was selected. The number of ChAT^+^ neurons in the FOV and the number of VGluT2^+^ or VGAT^+^ presynaptic terminals in direct contact with the soma surface of ChAT^+^ neurons were quantified. The average number of VGluT2^+^ or VGAT^+^ presynaptic terminals per soma surface of ChAT^+^ neuron in the FOV was then calculated, defined as the ratio of the total number of such presynaptic terminals to the total number of ChAT^+^ neurons.

### 2.19 Statistical analysis

All statistical analyses were performed using GraphPad Prism software (mean ± SD) were analyzed by Student's t-test or one-way ANOVA, followed by LSD (equal variances) or Dunnett's T3 (unequal variances) post hoc tests. Significance was defined as *P* < 0.05.

## 3 Results

### 3.1 Construction of the SCToid mimicking normal spinal cord tissue

**Fig. 1:**
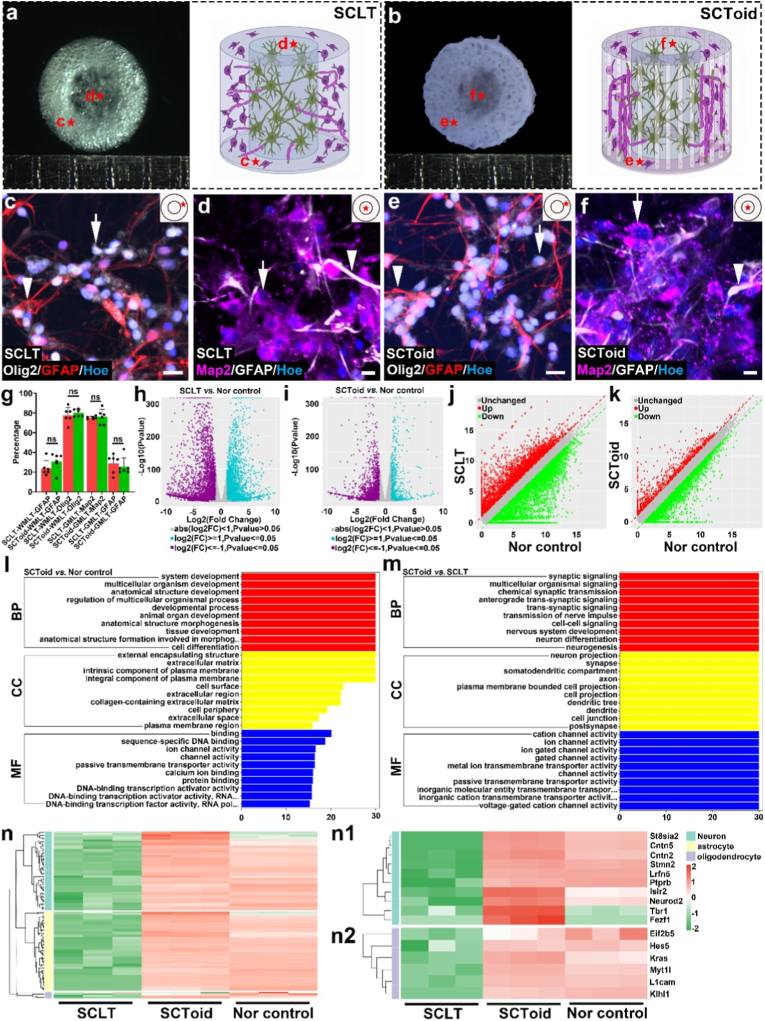
Tissue structure and cell identification. (a, b) SCLT and SCToid structures observed under a light microscope. (c, e) The images showed Olig2 (arrows) and GFAP (arrowheads) expression in differentiated WMLT cells. (d, f) The images showed Map2 (arrows) and GFAP (arrowheads) expression in differentiated GMLT cells. (g) Cell differentiation statistics (*n* = 6; ns: not significant). (h, i) Volcano plots showed differentially expressed genes (∣log2FC∣ ≥ 1) between the SCLT and Nor control groups (h) and between the SCToid and Nor control groups (i). (j, k) Scatter plots showed differentially expressed genes (∣log2FC∣ ≥ 1) between the SCLT and Nor control groups (j) and between the SCToid and Nor control groups (k). (l, m) GO enrichment bar chart shows the top 10 significantly enriched GO terms for differentially expressed genes (l, *P* ≤ 0.05), as well as the top 10 GO terms for genes significantly upregulated in the SCToid group compared with those in the SCLT group (m, *P* ≤ 0.05). (n) Cluster heatmap displays gene expression profiles. (n1) The top 10 genes with high neuronal expression in the SCToid group. (n2) The top 6 genes with high oligodendrocytes expression in the SCToid group. Hoechst 33342 (Hoe). Scale bars = 1 mm (a and b), 20 μm (c and e), and 10 μm (d and f).

CS was respectively shaped into WMLT and GMLT, and then they are assembled into SCLT (Fig. 1a). Scanning electron microscopy (SEM) revealed irregular pores (Supplementary Fig. 1a, a1), ranging in size from 10 to 150 μm. The CS-based GMLT scaffold for SCToid construction showed irregular pore distribution in cross-section (Fig. 1b; Supplementary Fig. 1b, b1), whereas the DON-based WMLT featured regularly aligned longitudinal channels with uniform 150–200 μm pores and small natural pores on channel walls (Supplementary Fig. 1b, b2, b3 and b4). Unlike the CS, which contained only collagen, the DON primarily contains LN and Col4, both of which are conducive to neural regeneration and development (Supplementary Fig. 1c). The seed cells were uniformly treated before they were seeded onto the WMLT and GMLT scaffolds. Nestin-positive (Nestin^+^) NSCs (Supplementary Fig. 1d) were induced to differentiate into OPCs, which were subsequently modified with the *CNTF* gene (Supplementary Fig. 1e) to construct the WMLT. The NSCs were modified with *NT-3 and TrkC* genes and then cocultured at a 1:1 ratio (Supplementary Fig. 1f) to construct the GMLT. Both the WMLT of the SCLT (Fig. 1c) and SCToid (Fig. 1e) were composed mainly of oligodendrocyte transcription factor 2-positive (Olig2^+^) oligodendrocytes, with a low proportion of glial fibrillary acidic protein-positive (GFAP^+^) astrocytes. In the GMLT, there was no significant difference in the proportion of microtubule-associated protein 2-positive (Map2^+^) neurons or GFAP^+^ astrocytes between the SCLT and SCToid groups (Fig. 1d–f and g). Cell differentiation assays showed that neither DON nor CS scaffolds significantly altered the trilineage differentiation potential of NSCs at the marker protein expression level.

To explore the similarities between the SCToid and normal spinal cord tissue as well as the differences between the SCToid and SCLT at the transcriptional level, we performed mRNA sequencing (RNA-seq) and comparisons of SCLT, SCToid and newborn rat spinal cord tissue (Nor control). The mRNA clustering heatmap, volcano plot and scatter plot of pairwise comparisons of differentially expressed mRNAs showed that the SCToid group had fewer differentially expressed mRNAs than the Nor control group (Fig. 1h–k), suggesting a closer resemblance in gene expression. The gene ontology (GO) enrichment bar chart showed that the differences in the topGO terms between the SCToid group and the Nor control group were mainly related to tissue development and cell differentiation, integral components of the plasma membrane and cell-ECM interactions (Fig. 1l). A significantly enriched GO bubble plot revealed upregulated genes, including genes related to brain-derived neural development, neurogenesis, and neuronal differentiation, in the SCToid group compared with the Nor control group (Supplementary Fig. 2). The GO enrichment bar chart showed that the SCLT group and the SCToid group exhibited differences in neuronal differentiation, synaptic signalling, neuron projection and voltage and ligand-gated ion channel activity (Fig. 1m). The mRNA clustering heatmap showed that compared with the SCLT, the SCToid significantly upregulated the expression of mRNAs involved in neuronal development (such as *Fezf1, Tbr1, Neurod2, Islr2, Cntn2, St8sia2, Cntn5, Stmn2, Ptprb, and Lrfn5*) and oligodendrocyte development (*such as Klhl1, L1cam, Myt1l, Eif2b5, Hes5, and Kras*), with expression levels closer to those of the Nor control (Fig. 1n, n1 and n2).

### 3.2 Integrin receptor-mediated cell-ECM interaction, differentiation, and functional development in the SCToid

**Fig. 2:**
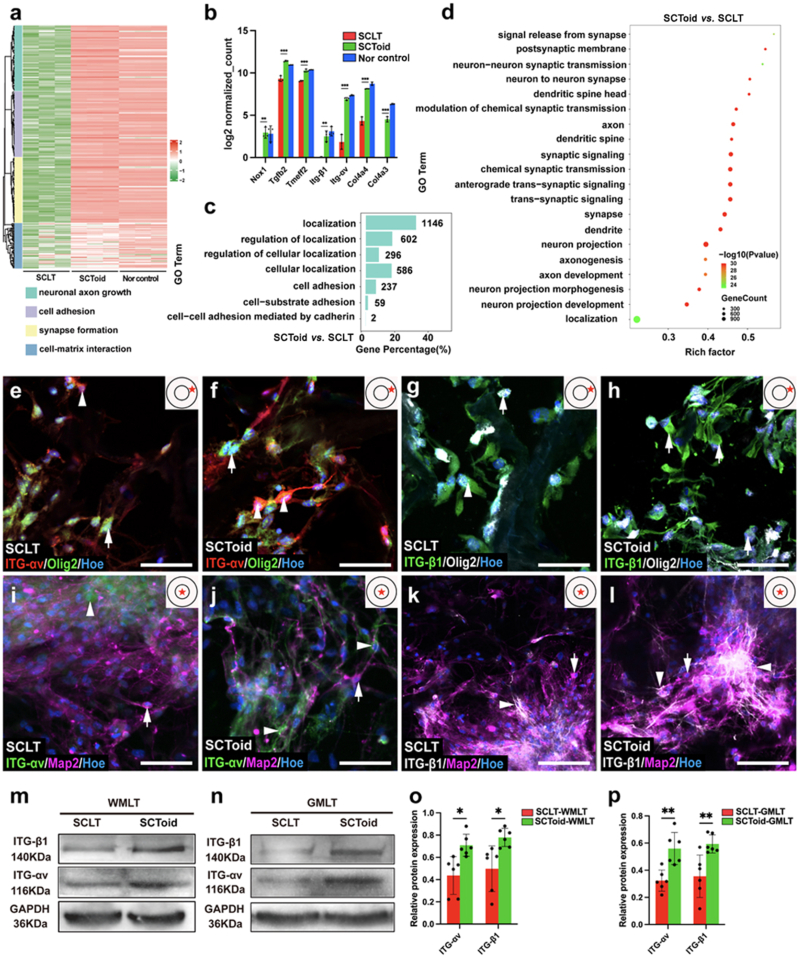
Gene analysis and differentiation characterization of the SCToid. (a) Cluster heatmap of genes involved in axon growth, cell adhesion, synapse formation, and cell-ECM interactions. (b) Expression of genes associated with integrin receptors and their synthesis regulation was analyzed. Data were input as base-2 logarithmic values to enable log normalization (*n = *3; ∗∗∗*P* < 0.001, ∗∗*P* < 0.01). (c) The topGO percentage bar plot shows the total number of upregulated genes. (d) The GO enrichment bubble plot shows the top 20 GO terms. (e−h, m, o) ITG-αv and ITG-β1 expression (arrowheads) in Olig2^+^ cells (arrows) in the WMLT of the SCLT and SCToid groups (e−h), representative protein immunoblots (m), and a bar graph quantifying relative expression levels (o, *n* = 6, ∗*P* < 0.05). (i−l, n, p) ITG-αv and ITG-β1 expression (arrowheads) in Map2^+^ neurons (arrows) in the GMLT of the SCLT and SCToid groups (i−l), representative protein immunoblots (n), and a bar graph quantifying relative expression levels (p, *n* = 6; ∗∗*P < *0.01). Scale bars = 20 μm (e−l).

The heatmap showed that compared with those in SCLT, the expression of mRNAs related to neuronal axon growth, cell adhesion, synapse formation, and cell-ECM interactions was significantly greater in the SCToid and Nor control (Fig. 2a). The expression of mRNAs related to integral components of the plasma membrane and cell-ECM interactions, such as *Itgb1, Itgav, Tmeff2, Tgfb2,* and *Nox1,* was significantly upregulated (Fig. 2b). In addition, the GO percentage bar plot (Fig. 2c) and the GO enrichment bubble plot (Fig. 2d) further showed that compared with those in SCLT, in the SCLT, the SCToid exhibited a significant increase in cell-ECM interactions, as well as in the expression of mRNAs related to neuronal projection, morphogenesis, development, localization and synaptic transmission (Supplementary Fig. 3).

To further test this hypothesis, we investigated the expression levels of ITG-αv and ITG-β1 in SCToid and SCLT 7 days after the assembly of WMLT and GLML *in vitro*. Compared with those in the WMLT of the SCLT, the levels of ITG-αv and ITG-β1 in Olig2^+^ oligodendrocytes were significantly higher in the WMLT of the SCToid (Fig. 2e−h, m and o); and compared with those in the GMLT of the SCLT, the levels of ITG-αv and ITG-β1 in Map2^+^ neurons were also higher in the GMLT of the SCToid (Fig. 2i−l, n and p), suggesting that the microenvironment provided by the DON may induce upregulation of integrin receptor expression in the SCToid and support neuronal differentiation and development.

### 3.3 Integrin receptor-mediated cell-ECM interactions regulate neuronal differentiation and maturation in the SCToid

**Fig. 3:**
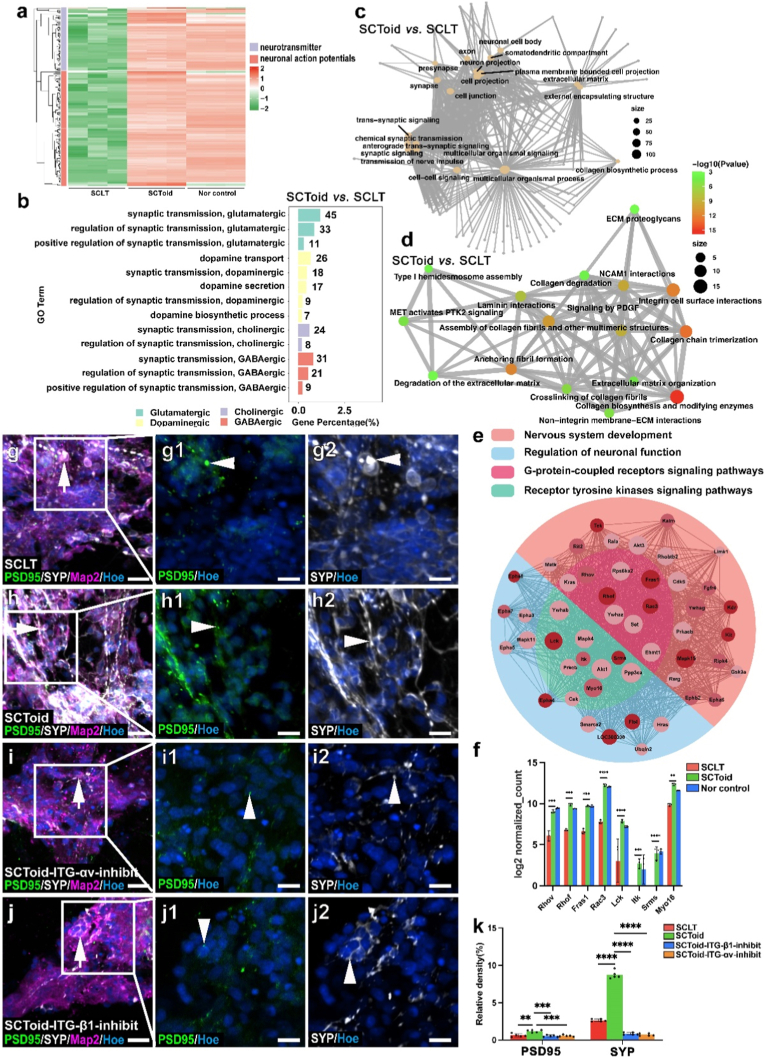
Detection of cell-ECM interactions. (a) Clustering heatmap based on differentially expressed genes. (b) Bar chart showing the percentage of genes associated with each GO term. (c) Network diagram of enriched gene–GO term associations; node size represents the number of candidate genes in each functional category. (d) Network visualization of the top 20 GO categories and their overlap with upregulated differentially expressed genes, with node sizes representing the total number of candidate genes belonging to the GO category and colour indicating -log10 (*P* value). (e) Protein-protein interaction (PPI) network of the top 50 upregulated genes in the SCToid *vs.* the SCLT groups; node size indicates number of interaction partners. (f) Expression of genes in G protein-coupled receptor signalling pathways and receptor tyrosine kinase-related signalling pathways. Data were input as base-2 logarithmic values to enable log normalization (*n = *3; ∗∗∗∗*P* < 0.0001, ∗∗∗*P* < 0.001, ∗∗*P* < 0.01). (g−h2) PSD95 and SYP expression in Map2^+^ neurons (arrows). (i–j2) Reduced PSD95 and SYP expression (arrowheads) in the SCToid following ITG-αv (i–i2) or ITG-β1 (j–j2) blockade. (k) Bar graph of PSD95 and SYP fluorescence intensity (*n* = 5; ∗∗*P* < 0.01, ∗∗∗*P* < 0.001, ∗∗∗∗*P* < 0.0001). Scale bars = 20 μm (g−j) and 10 μm (g1−j1 and g2−j2). (For interpretation of the references to colour in this figure legend, the reader is referred to the Web version of this article.)

The heatmap showed significantly greater mRNA levels of neurotransmitters and neuronal action potentials in the SCToid and Nor control groups than in the SCLT group (Fig. 3a). The bar chart, which showed the percentage ranking of upregulated mRNAs, displayed enrichment for glutamatergic, dopaminergic, cholinergic, and GABAergic neurons in the SCToid versus SCLT comparison. Importantly, the level of excitatory neurotransmitter mRNAs in the SCToid group was significantly greater than that in the SCLT group, suggesting that the SCToid group contains a greater proportion of excitatory neurons (Fig. 3b). To further reveal the key regulatory networks involved, we analyzed the interactions of upregulated mRNAs, which showed a strong correlation among the ECM, neuron projection and synaptic activity in the SCToid vs. SCLT comparison (Fig. 3c), and in the SCToid vs. Nor control comparison (Supplementary Fig. 4). The results also strongly interacted between the ECM (especially the LN and Col4) and integrin receptor (Fig. 3d). Based on protein interaction analysis using the STRING database of the top 50 significantly upregulated genes in the SCToid vs. SCLT comparison, the results suggest that the microenvironment of the ECM created by the SCToid may play a critical role in nervous system development and the regulation of neuronal function by upregulating the expression of integrin receptor and activating their associated G protein-coupled receptor signalling pathways and receptor tyrosine kinase-related signalling pathways (Fig. 3e and f).

During the *in vitro* co-culture process, the neurons in the GMLT of SCToid extended many neurites into the WMLT (Supplementary Fig. 5), and were influenced by the ECM of DON in the white matter region, resulting in up-regulation of ITG-αv and ITG-β1 expressions. As a result, neurons in the GMLT exhibited the enhanced expression of synaptic-related proteins PSD95 and SYP under the influence of DON (Fig. 3g, h and k). When ITG-αv and ITG-β1 were inhibited, the expression levels of PSD95 and SYP in neurons within the GMLT of SCToid correspondingly decreased (Fig. 3i, j and k).

### 3.4 Transplanted SCToid promotes axonal regeneration and myelination in the injury/graft site of spinal cord

**Fig. 4:**
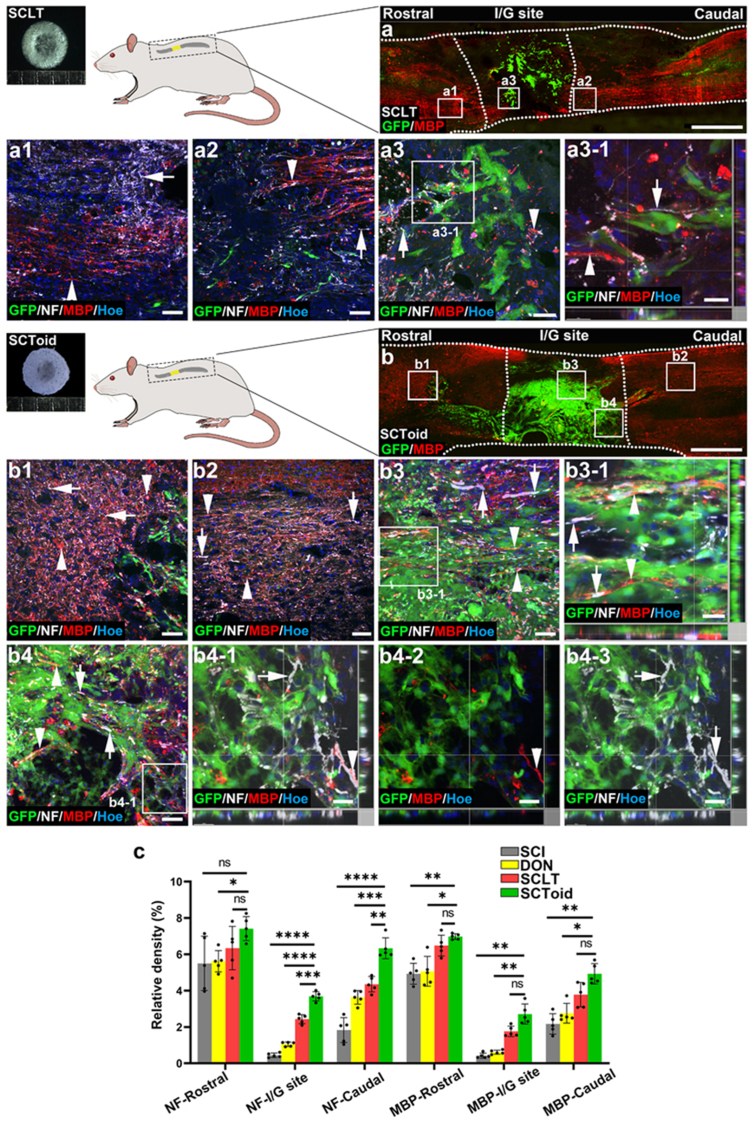
Neural regeneration and integration of SCToid grafts with the host spinal cord. (a) Sagittal spinal cord section showed NF^+^ axons (arrows) and MBP^+^ myelin sheaths (arrowheads) in the areas rostral (a1) and caudal (a2) to/in the injury/graft site (a3). (a3-1) The boxed area in (a3) showed NF^+^ axons (arrow) in contact with GFP^+^ cells, with no MBP^+^ myelin sheath wrapping. (b) Sagittal spinal cord section showed NF^+^ axons (arrows) and MBP^+^ myelin sheaths (arrowheads) in the areas rostral (b1) and caudal (b2) to/in the injury/graft site (b3, b4); regenerated NF^+^ axons wrapped by MBP^+^ myelin sheaths on the dorsal graft side (b3, b3-1); and host/donor-derived NF^+^ axons and MBP^+^ myelin sheaths on the ventral graft side (b4, b4-1–b4-3). (c) Relative NF and MBP expression levels (*n* = 5; ∗*P* < 0.05, ∗∗*P* < 0.01, ∗∗∗*P* < 0.001, ∗∗∗∗*P* < 0.0001; ns: not significant). Scale bars = 1 mm (a and b), 50 μm (a1−a3 and b1−b4) and 20 μm (a3-1, b3-1, b4-1, b4-2, and b4-3).

Herein, SCLT and SCToid were transplanted into the spinal cord injury site, followed by a comparative assessment of their effects on spinal cord neural regeneration and tissue repair. Two months after transplantation, GFP^+^ donor cells survived robustly in the SCLT and SCToid groups (Fig. 4a and b). The regeneration of NF^+^ axons and the formation of MBP^+^ myelin sheaths were examined. The results showed that many NF^+^ axons surrounded by MBP^+^ myelin sheaths appeared in the areas rostral and caudal to the injury/graft site of the spinal cord in the SCLT group (Fig. 4a1 and a2), but in the injury/graft site, NF^+^ axons surrounded by MBP^+^ myelin sheaths were significantly less abundant (Fig. 4a3 and a3-1). There were more NF^+^/MBP^+^ myelinated axons in the areas rostral and caudal to the injury/graft site in the SCToid group than in the SCLT group (Fig. 4b1 and b2). Importantly, in the sagittal section sides (the dorsal aspect is positioned superiorly and the ventral aspect inferiorly), many NF^+^ axons were detected in the injury/graft site, which extended directly to the dorsal aspect and were surrounded by MBP^+^/GFP^+^ transplanted oligodendrocytes in the WMLT area of the SCToid (Fig. 4b3 and b3-1). Moreover, host NF^+^ axons and transplanted NF^+^/GFP^+^ axons also appeared in the GMLT area of the SCToid (Fig. 4b4, b4-1, b4-2 and b4-3). In comparison, although there were more NF^+^/MBP^+^ myelinated axons in the areas rostral and caudal to/in the injury/graft site in the DON group than in the SCI group (Supplementary Fig. 6), these values were still significantly lower than those in the SCLT and SCToid groups (Fig. 4c and Supplementary Fig. 7). In the DON group, a few regenerated NF^+^ axons were observed only in the injury/graft site, but they were rarely surrounded by MBP^+^ myelin sheaths (Supplementary Fig. 6).

To further observe whether there was a difference in synapse formation in the SCLT and SCToid groups and whether this difference was related to upregulated integrin receptor expression *in vitro*, we performed coexpression analysis of SYP and integrin receptor in the injury/graft site. Compared with those in the SCLT group, the levels of SYP and ITG-αv expression were significantly higher in the SCToid group (Supplementary Fig. 8a, b and e), and the levels of SYP and ITG-β1 expression were also significantly greater in the SCToid group. In the SCToid group, more SYP^+^ presynaptic terminals were distributed around GFP^+^ cells (Supplementary Fig. 8c, d and e). At the 8-week post-transplantation time point in the SCLT and SCToid groups, GFAP was used to visualize glial scar formation, while CD68 was employed to label inflammatory cells. The results demonstrated that no significant GFAP^+^ astrocyte scar or CD68^+^ macrophage/microglia infiltration was observed at the graft–host interface in either group (Supplementary Fig. 8f–i).

### 3.5 Improvement of locomotor function and neural pathway efficiency following SCToid transplantation combined with TNES

**Fig. 5:**
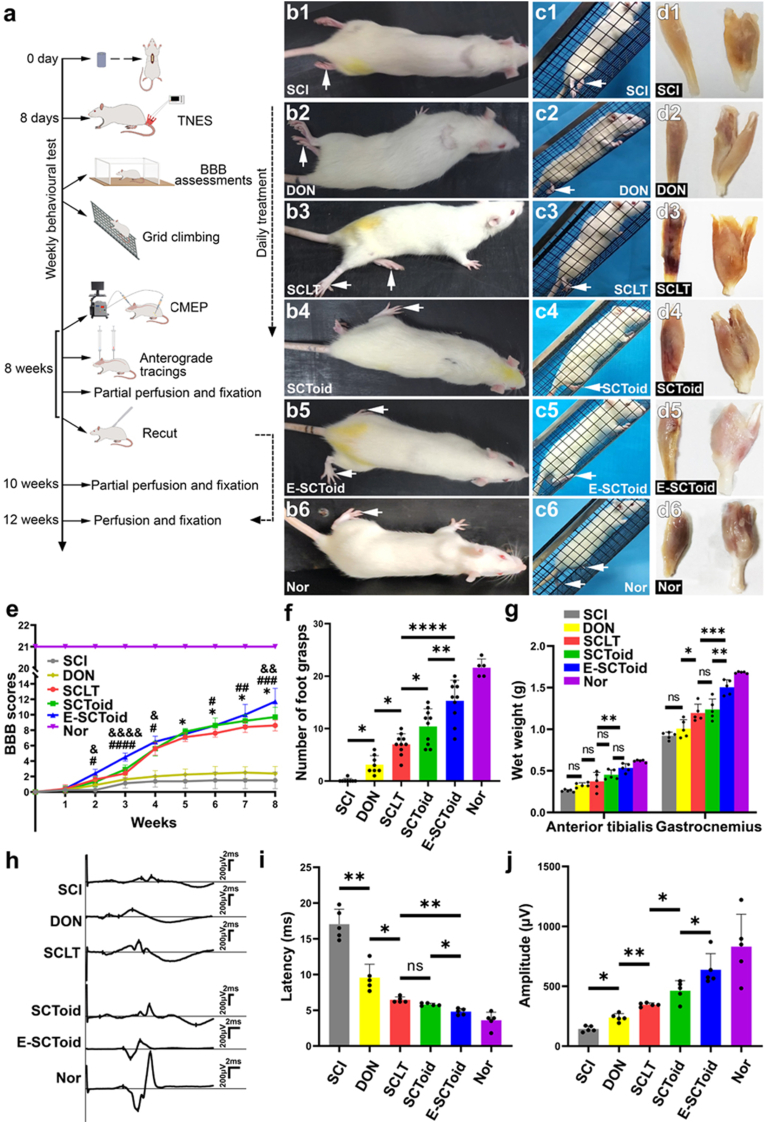
Behavior, electrophysiology and skeletal muscle atrophy analysis. (a) Overall flowchart of the animal experiment. (b1−b6) Open-field images of each group demonstrating recovery of hindlimb joint movement patterns and weight-bearing capacity. (c1−c6) Grid climbing test for each group, showing hindlimb placement and climbing behavior on the grid. (d1−d6) Images of the anterior tibialis and gastrocnemius muscles in each group. (e) BBB scores for each group (*n* = 10; ^#^ and ^&^ indicate statistical significance for comparisons of the E-SCToid group with the SCLT and SCToid groups, respectively, and ∗ indicates statistical significance for the comparison of the SCToid group with the SCLT group; ^#^*P* < 0.05, ^##^*P* < 0.01, ^###^*P* < 0.001, ^####^*P* < 0.0001, ^&^*P* < 0.05, ^&&^*P* < 0.01, ^&&&&^*P* < 0.0001, ∗*P* < 0.05). (f) Number of foot grasps on the grid for each group (*n* = 8; ∗*P* < 0.05, ∗∗*P* < 0.01, ∗∗∗∗*P* < 0.0001). (g) Wet weights of the anterior tibialis and gastrocnemius muscles in each group (*n* = 5; ∗*P* < 0.05, ∗∗*P* < 0.01, ∗∗∗*P* < 0.001; ns: not significant). (h−j) CMEP curve (h) and latency (i) and peak-to-peak amplitude (j) of each group of rats (*n* = 5; ∗*P* < 0.05, ∗∗*P* < 0.01; ns: not significant).

To assess whether axon regeneration and myelination could improve rat hindlimb movement, we performed the BBB open-field locomotor test, the modified inclined grid climbing test, and the skeletal muscle atrophy test (Fig. 5a−g, Supplementary Video 2 showing open-field locomotor test and Supplementary Video 3 showing grid climbing test). The results showed that rats in the SCI group maintained BBB scores consistently below 3 throughout the 2-month observation period, and occasional large-joint movements were observed during the inclined grid climbing test (Fig. 5b1, c1 and e). In the DON group, rats exhibited movement across three joints, but the BBB score did not exceed 5, and occasional grid climbing actions were observed (Fig. 5b2, c2 and e). In the SCLT group, rats exhibited weight-bearing hindlimb movement but predominantly displayed scraping-like movements—with the dorsal surface of the foot contacting the ground—and achieved a BBB score approaching 9 (Fig. 5b3 and e). During the grid climbing test, the number of foot grasps on the grid frame in the SCLT group was significantly greater than in the DON group (Fig. 5c3 and f). The SCToid group showed further improvement: the BBB score approached 10, with frequent hindpaw support during weight-bearing locomotion and frequent foot grasping of the grid frame during the grid climbing test (Fig. 5b4, c4, e and f). In the E-SCToid group—where TNES was combined with SCToid transplantation—the BBB score approached 12 (Fig. 5b5 and e). In addition to weight-bearing walking with the hind paws, frequent coordination movements of the forelimbs and hindlimbs were observed (Supplementary Video 2 showing open-field locomotor test), and during the grid climbing test, the rats successfully climbed the grid with coordinated movement of the forelimbs and hindlimbs (Fig. 5c5 and f; Supplementary Video 3 showing grid climbing test). TNES exerted a partial ameliorative effect on the BBB locomotor scores in the E-SCI and E-SCLT groups. However, at 8 weeks post-treatment, scores in these groups remained significantly lower than those in the E-SCToid group, with no significant recovery of coordinated movement function (Supplementary Fig. 9). The reduction in skeletal muscle atrophy was consistent with the improvement in rat movement. At 8 weeks, there were no significant differences in the body weights of rats in each group, but the wet weights of the gastrocnemius muscles in the E-SCToid group were significantly greater than those in the SCToid group, approaching those in the Normal group (Nor group) (Fig. 5d1−d6 and g).

The results of the CMEP test showed that the SCToid group had a significantly greater amplitude and shorter latency than did the SCI, DON and SCLT groups (Fig. 5h−j), which consistent with the axon regeneration, myelination and behavioral recovery results. Interestingly, the E-SCToid group displayed greater amplitudes and shorter latencies than did the SCToid group (Fig. 5h−j), indicating an increase in the efficiency of neural pathways to relay brain-derived neural information.

### 3.6 Regenerated host axons exhibit integration with SCToid neurons following TNES

**Fig. 6:**
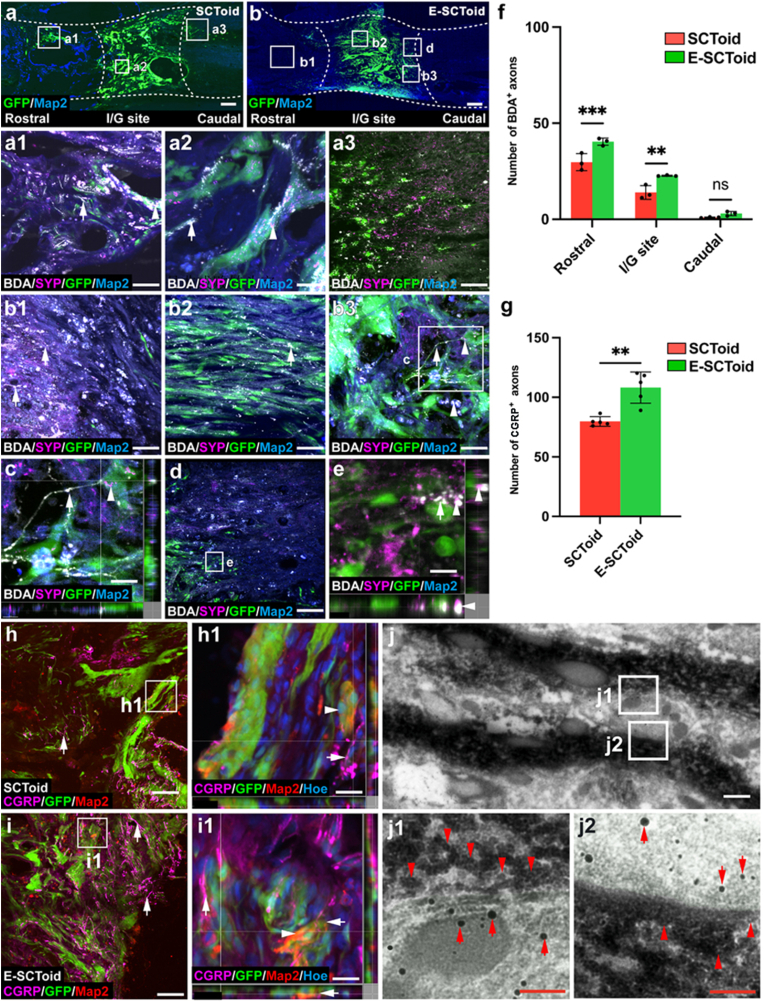
Reconstruction of ascending and descending neural pathways. (a–a3) Sagittal spinal cord section from the SCToid group showed that BDA^+^ axons (arrows) regenerated extensively in the area rostral to/in the injury/graft site (a1, a2), where they closely contacted GFP^+^/Map2^+^ cells (arrowheads). In contrast, few BDA^+^ axons were observed in the area caudal to the injury/graft site (a3). (b–e) In the E-SCToid group, more BDA^+^ axons regenerated in the area rostral to/in the injury/graft site compared with the SCToid group (a1). Many BDA^+^ axons (arrow) regenerated into the dorsal side (b2) and ventral side (b3) of the injury/graft site, where they closely contacted GFP^+^/Map2^+^ cells (arrowhead), and these contact site expressed SYP (c). BDA^+^ axons were detected in the caudal region of the injury/graft site (d and arrow, e), where they formed close contacts with transplanted neurons; these contact sites exhibited SYP expression (arrowheads, e). (f) Quantification of BDA^+^ axons (*n* = 3; ∗∗*P* < 0.01, ∗∗∗*P* < 0.001; ns: not significant). (g) Quantification of CGRP^+^ axons (*n* = 5; ∗∗*P* < 0.01). (h–i1) CGRP^+^ axons (arrows) regenerated into the injury/graft site and contacted Map2^+^ transplanted neurons (arrowheads). (j–j2) CGRP^+^ axons (DAB-stained) formed synapse-like structures characterized by synaptic vesicles (arrowheads), axonal membrane thickening, and acting as presynaptic components in contact with nanogold^+^ transplanted neurons (arrows). Scale bars = 500 μm (a and b), 50 μm (a1, a3, b1−b3, and d), 20 μm (a2, h1, and i1), 10 μm (c), 5 μm (e), 100 μm (h and i), 1 μm (j), and 200 nm (j1 and j2).

Given that the neuroregulatory effects of TNES rely on sufficient neural regeneration and structural repair of the damaged spinal cord tissue, we further validated the synaptic connectivity between regenerated corticospinal tract (CST) axons and SCToid neurons following TNES intervention. Specifically, we performed BDA tracing in the SMC of rats in the SCToid and E-SCToid groups (Supplementary Fig. 10). The results showed that the BDA^+^ CST axons were able to regenerate to the injury/graft site (Fig. 6a–a1 and a2); additionally, a few BDA^+^/SYP^+^ axons were in close contact with GFP^+^/Map2^+^ neurons (Fig. 6a2) in the SCToid group. However, none of the BDA^+^ axons can regenerate beyond the injury/graft sites to the caudal regions in the SCToid group (Fig. 6a3). There were more BDA^+^ axons in the injury/graft site in the E-SCToid group than in the SCToid group (Fig. 6b−f). In the dorsal (WMLT) area of the E-SCToid group, some straight BDA^+^ axons were observed (Fig. 6b2), while in the ventral (GMLT) area and in the grafted caudal area of the E-SCToid group, the BDA^+^/SYP^+^ axons appeared in close contact with the GFP^+^/Map2^+^ neurons (Fig. 6b3 and c). The BDA^+^ axons were able to regenerate to caudal regions of the injury/graft sites (Fig. 6d); additionally, a few BDA^+^/SYP^+^ axons were in close contact with GFP^+^/Map2^+^ neurons (Fig. 6e) in the E-SCToid group. These findings suggest that the CST is more likely to regenerate and form synaptic connections with SCToid neurons under the influence of TNES**.**

To further investigate whether TNES promotes the synaptic connectivity of regenerated sensory afferent axons with SCToid neurons, we analyzed CGRP^+^ axons in the injury/graft site in the SCToid and E-SCToid groups. Compared with those in the SCToid group, more CGRP^+^ axons in the E-SCToid group were able to regenerate to the injury/graft site (Fig. 6g−i1; Supplementary Fig. 11). IEM revealed that CGRP^+^ presynaptic terminals exhibited synaptic connectivity with GFP^+^ neurons in the E-SCToid group (Fig. 6j−j2).

### 3.7 SCToid neurons and sensory afferent axons integrate with CPG-regulated motor neural circuits

**Fig. 7:**
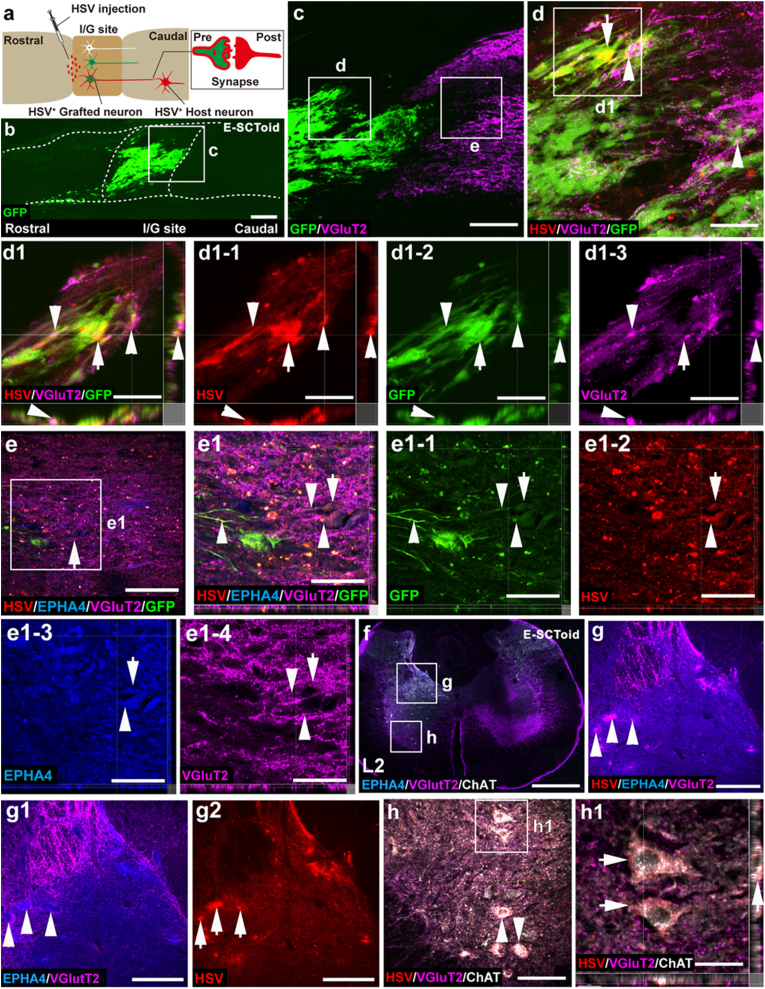
HSV and CTB tracing. (a) Schematic of transmonosynaptic HSV anterograde tracing. (b) The sagittal spinal cord sections from the E-SCToid group. (c) The boxed area in (b) showed the distribution of GFP^+^ cells and the regeneration of VGluT2^+^ axons. (d) The boxed area in (c) showed that some GFP^+^ cells infected with HSV exhibited yellow fluorescence (arrows, d1), which were in close contact with the regenerated VGluT2^+^ axons (arrowheads, d1-2, d1-3), and that some GFP^+^ cells differentiated into VGluT2^+^ neurons (arrowhead, d1, d1-2, d1-3). (e−e1-4) The boxed area in (c) showed GFP^+^ cells extending VGluT2^+^ axons (arrowheads, e1, e1-4) that form close contacts with host neurons co-expressing HSV, EPHA4, and VGluT2 (arrows, e1, e1-2–e1-4). (f, g−g2) The boxed area in (f) showed EPHA4^+^/VGluT2^+^ neurons (arrowheads, g and g1), which exhibited red fluorescence (arrows) after being infected with HSV (g2). (h, h1) Higher magnification image of the boxed area in (f) showed motor neurons in the ventral horn expressing ChAT (arrowheads), and additional magnification reveals some HSV^+^/VGluT2^+^ axon terminals (arrows) on the soma surface of these motor neurons (h1). Scale bars = 500 μm (b and f), 200 μm (c), 50 μm (d, e, g, g1−g2, and h), 20 μm (d1, d2, and e1), and 15 μm (h1). (For interpretation of the references to colour in this figure legend, the reader is referred to the Web version of this article.)

A key question explored in this study is how SCToid neurons transmit neural information to motor neural circuits in the area caudal to the injury/graft site after they receive and interpret descending motor and ascending sensory information. Anterograde transmonosynaptic tracing was employed to identify the host neurons in the area caudal to the injury/graft site that were directly innervated by the SCToid neurons. SCToid neurons were transfected with AAV-helper, and one week before the rats were perfused, HSV was injected into the area rostral to the injury/graft site (Fig. 7a and b), which was able to effectively infect SCToid neurons (presynaptic neurons) and transmit to host neurons (postsynaptic neurons) in the area caudal to the injury/graft site. The results showed that GFP^+^/NeuN^+^ neurons were successfully infected with HSV (red fluorescence) and exhibited yellow fluorescence (Fig. 7b−d and d1−d1-3; Supplementary Fig. 12).

In the injury/graft site, some regenerated VGluT2 axons were in contact with HSV^+^/GFP^+^ neurons (Fig. 7d and d1−d1-3), and some GFP^+^ neurons were VGluT2-positive (Fig. 7d1-2 and d1-3). Notably, we found that HSV could be transmitted to EPHA4^+^/VGluT2^+^ neurons in the area caudal to the injury/graft site via HSV^+^/GFP^+^/VGluT2^+^ neurons (Fig. 7e and e1−e1-3). Interestingly, some HSV^+^/EPHA4^+^/VGluT2^+^ interneurons located in the median region clearly appeared in the cross-sections of the L2 and L3 spinal cord segment (Fig. 7f and g−g2; Supplementary Fig. 13). In addition, we also observed that some HSV^+^/VGluT2^+^ presynaptic terminals derived from EPHA4^+^/VGluT2^+^ subtype of CPG interneurons were distributed around the soma surface of ChAT^+^ motor neurons located in the lateral part of the L2 ventral horn (Fig. 7h and h1).

To investigate whether CPG interneurons in the spinal L2 segment can be innervated by sensory afferent axons derived from the tail nerve, we injected the CTB into the tail nerve. The results showed CTB positive (CTB^+^) afferent axons in near median region in cross-sections of the L2 and L4 segments (Supplementary Fig. 14), and some CTB and vesicular glutamate transporter 1 (VGluT1) double-positive (CTB^+^/VGluT1^+^) axonal terminals derived from afferent axons were distributed around the soma surface of EPHA4^+^/VGluT2^+^ CPG interneurons (Supplementary Fig. 14a1, b, c1 and c2). The results revealed that afferent VGluT1^+^ axons derived from the tail nerve are able to innervate CPG interneurons under the influence of TNES.

### 3.8 Restoring distribution of excitatory/inhibitory synaptic terminals innervating spinal cord interneurons in the area caudal to the injury/graft site

**Fig. 8:**
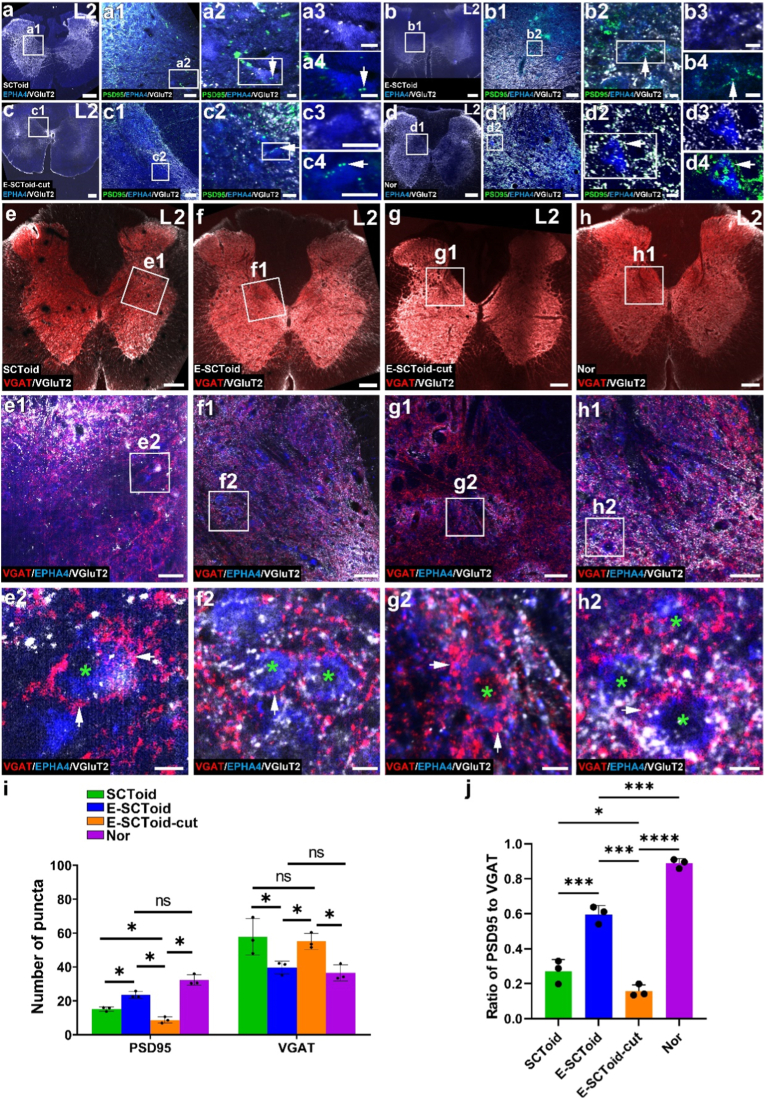
Synaptic terminal distribution patterns. (a−d4) Showing PSD95^+^ puncta (arrows) on the soma surface of EPHA4^+^/VGluT2^+^ neurons. (e−h2) Showing VGAT^+^ puncta (arrows) on the soma surface of EPHA4^+^/VGluT2^+^ neurons (asterisks). (i) The number of PSD95^+^ and VGAT^+^ puncta on the soma surface of EPHA4^+^/VGluT2^+^ neurons in the L2 segment was quantified for each group (*n* = 3; ∗*P* < 0.05; ns: not significant). (j) The ratio of PSD95^+^ to VGAT^+^ puncta on the soma surface of EPHA4^+^/VGluT2^+^ neurons was quantified (*n* = 3; ∗*P* < 0.05, ∗∗∗*P* < 0.001, ∗∗∗∗*P* < 0.0001). Scale bars = 200 μm (a−h), 50 μm (a1−h1), and 10 μm (a2−a4, b2−b4, c2−c4, d2−d4, and e2−h2).

To investigate the effects of E-SCToid on neural pathway reconstruction, we analyzed TNES-mediated modulation of synaptic plasticity in the L2 spinal cord segments (Fig. 8). The results showed that some postsynaptic density protein 95-positive (PSD95^+^) puncta (i.e. excitatory postsynaptic structures, that form the excitatory synapses together with the presynaptic terminals) were located in the EPHA4^+^/VGluT2^+^ interneurons in the median region in cross-sections of the L2 segment in the SCToid, E-SCToid, E-SCToid-cut and Nor groups (Fig. 8a−d4). Compared with the SCToid group, the number of PSD95^+^ puncta in the L2 segment was significantly greater in the E-SCToid group and closer to that in the Nor group (Fig. 8b−b4, d−d4 and i). However, a significant decrease in the number of PSD95^+^ puncta was observed in the EPHA4^+^/VGluT2^+^ subtype of CPG interneurons in the E-SCToid-cut group (Fig. 8c−c4 and i), accompanied by loss of motor function (Supplementary Video 4 showing open-field locomotor test). The results also showed that VGAT^+^ presynaptic terminals were distributed around the soma surface of the EPHA4^+^/VGluT2^+^ subtype of CPG interneurons in the SCToid, E-SCToid, E-SCToid-cut, and Nor groups (Fig. 8e−h2). The number of VGAT^+^ puncta in the E-SCToid group was lower than that in the SCToid and E-SCToid-cut groups and was closer to that in the Nor group (Fig. 8i). The ratio of PSD95^+^ to VGAT^+^ puncta (excitatory/inhibitory synaptic ratio) in the EPHA4^+^/VGluT2^+^ subtype of CPG interneurons was comparable between the E-SCToid group and the Nor group (Fig. 8j).

### 3.9 Improving distribution of excitatory/inhibitory synaptic terminals innervating spinal cord motor neurons

**Fig. 9:**
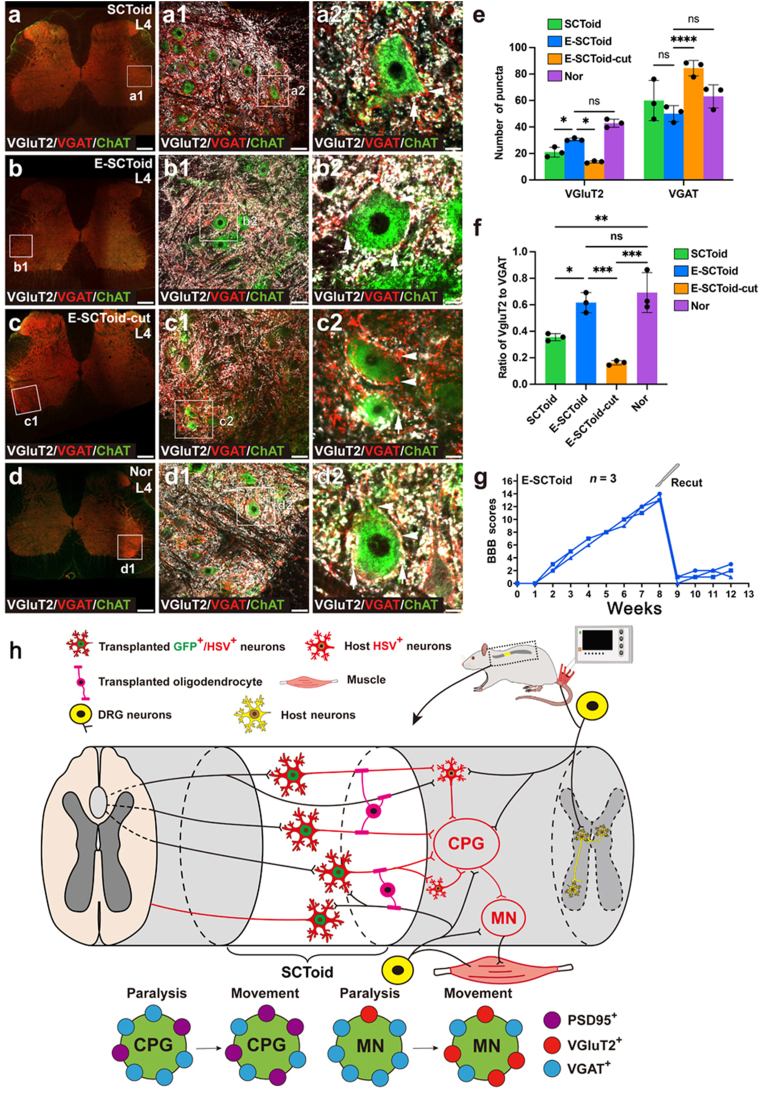
Synaptic terminals on the soma surface of motor neurons. (a−d2) Visualization of ChAT^+^ motor neurons, VGluT2^+^ puncta (arrows), and VGAT^+^ puncta (arrowheads). (e) The number of VGluT2^+^ and VGAT^+^ puncta on the soma surface of L4 motor neurons was quantified for each group (*n* = 3; ∗*P* < 0.05, ∗∗∗∗*P* < 0.0001; ns: not significant). (f) The ratio of VGluT2^+^ to VGAT^+^ puncta was quantified (*n* = 3, ∗*P* < 0.05, ∗∗*P* < 0.01, ∗∗∗*P* < 0.001). (g) BBB score. (h) Schematic of how SCToid transplantation combined with TNES promotes the reconstruction of spinal cord neural pathways and restores the excitatory/inhibitory balance of sensorimotor neural circuits. Scale bars = 200 μm (a−d), 50 μm (a1−d1), and 10 μm (a2−d2).

In the L4 segment, VGluT2^+^ presynaptic terminals (i.e. VGluT2^+^ puncta, that are excitatory presynaptic structures) and VGAT^+^ presynaptic terminals (VGAT^+^ puncta) were distributed around the soma surface of ChAT^+^ motor neurons in the SCToid, E-SCToid, E-SCToid-cut, and Nor groups (Fig. 9a−f). The number of VGluT2^+^ puncta was greater in the E-SCToid group than in the SCToid group and was closer to that in the Nor group (Fig. 9e). Nevertheless, a significant decrease in the number of VGluT2^+^ puncta distributed around the soma surface of ChAT^+^ motor neurons was observed in the E-SCToid-cut group (Fig. 9e), accompanied by locomotor dysfunction (Supplementary Video 4 showing open-field locomotor test) and a decreased BBB score (Fig. 9g). Similarly, VGAT^+^ puncta distributed around the soma surface of ChAT^+^ motor neurons were detected in the SCToid, E-SCToid, E-SCToid-cut and Nor groups (Fig. 9a−f). The number of VGAT^+^ puncta in the L4 segment did not differ among the SCToid, E-SCToid and Nor groups (Fig. 9e). However, an obvious increase in the number of VGAT^+^ puncta around the soma surface of ChAT^+^ motor neurons was observed in the E-SCToid-cut group (Fig. 9e). The ratio of VGluT2 to VGAT (excitatory/inhibitory) synaptic puncta on the soma surface of motor neurons in the E-SCToid group was close to that in the Nor group (Fig. 9f). These findings may contribute to the recovery of voluntary movement of the paralyzed hindlimbs (Fig. 9h).

## 4 Discussion

In this study, we constructed an SCToid model by simulating the developmental microenvironment and structural characteristics of the spinal cord and found that upregulation of integrin receptor expression in SCToid neurons is an important factor for efficient reconstruction of neural pathways. SCToid transplantation and TNES promote functional integration of regenerated CST and sensory afferent axons with SCToid neurons. SCToid transplantation requires concurrent TNES to restore the fine balance between excitation and inhibition in CPG-regulated sensorimotor neural circuits, thereby improving voluntary movement in paralyzed hindlimbs.

### 4.1 Therapeutic value of SCToid transplantation in spinal cord repair

Compared with the incomplete SCI model, the transected SCI model is ideal for studying functional reconstruction driven by axonal regeneration and neuronal relayer [[35], [36], [37], [38], [39]]. The neuronal relayer is a structural and functional unit in the injury/graft site of spinal cord and is mainly derived from neural stem cells. After receiving and integrating multiple input electrical signals, these neurons are able to conduct bioelectrical signals to postsynaptic neurons and adjust their activity through the opening of voltage-gated ion channels in the presynaptic membrane [38]. Studies using transected SCI models have suggested that the key to restoring voluntary locomotor function lies in transplanting neurons that can relay brain-derived neural information to host neurons in the area caudal to the lesion [23,36,37]. The results from our previous research have indicated that co-transplantation of stem cell-derived neurons and myelin-forming cells reestablishes neural pathways more efficiently [31]. Based on this concept, we developed a SCLT consisting of a WMLT (composed of oligodendrocytes) and a GMLT (composed of neurons), which has been showed to partially re-establish the neural pathway of descending spinal projection neurons and enhance voluntary locomotor function [8]. However, the CS scaffold cannot provide longitudinal channels for the straight growth of axons. Based on studies using DON to construct WMLT to replace damaged spinal white matter [25], we constructed an SCToid for replacing damaged spinal cord tissue.

The SCToid constructed using DON as the WMLT scaffold features a brain-derived neural ECM microenvironment and longitudinal channels that guide axonal growth [25,40], and it differs from central nervous system organoids, which self-organize from the developmental stage and are primarily used for modeling neurological diseases and drug screening [41,42]. The SCToid culture directly induces the differentiation of NSCs into OPCs to form WMLT or into neurons and astrocytes to form GMLT; it prevents the heterogeneity associated with NSC self-organized development. Interestingly, RNA-seq analysis revealed that the proportions of synapse formation and neural network functional characteristics in the SCToid were close to those in normal spinal cord tissue from newborn rats, and the potential for synapse formation is associated with increased neuronal integrin receptor expression, which may be induced by the enrichment of LN and Col4 (axon growth path molecules) in DON [29,43]. The SCToid can be used for transplantation to replace large-volume spinal cord tissue defects (≥2 mm); it not only restores the structure and cellular composition of spinal cord white and gray matter but also supports functional neural pathway reconstruction. We speculate that, relative to current transplantation strategies, the SCToid could yield superior regeneration and myelination of ascending and descending axons, alongside enhanced synaptic connectivity—critical prerequisites for reconstructing spinal cord neural pathways.

### 4.2 Necessity of reconstructing spinal cord neural pathways and activating sensorimotor neural circuits

Two months after transplantation, histological analysis showed that both SCToid and SCLT integrated well with the host spinal cord, with no significant inflammatory cell accumulation or glial scarring at the interface. The histological analysis further validated our hypothesis that, compared with SCLT, SCToid more effectively promoted straight axonal regeneration and myelination. Importantly, upregulation of integrin receptor expression in transplanted SCToid neurons significantly enhanced their capacity to form synaptic connections with regenerated axons, which was consistent with RNA-seq results. In addition, rats in the SCToid group showed significantly improved CMEP amplitudes and recovery of hindlimb weight-bearing movement compared with those in the SCLT group. These findings suggest that in future strategies for spinal cord injury repair, it is necessary to promote not only axonal regeneration and myelination but also synaptic connectivity by upregulating integrin receptor expression in transplanted neurons [44,45]. However, another key bottleneck after neural pathway reconstruction is functional silencing of sensorimotor neural circuits below the lesion site and consequent muscle atrophy in paralyzed limbs [46,47]. To address this issue, we first validated that SCToid enables more effective reconstruction of spinal cord neural pathways. Building on this finding, we further investigated the efficacy and underlying mechanisms of combined SCToid transplantation and TNES in promoting voluntary locomotor recovery.

The results of our previous research suggested that TNES applied to rats with transected SCI activated the CPG-regulated sensorimotor neural circuits and effectively alleviated hindlimb muscle atrophy [24,48]. The CPG is a neural network composed of locomotion-generating interneurons that coordinate to activate distinct motor neuron pools and produce alternating left–right hindlimb movement [26,49]. Even in the early stage of complete transection of the rat T10 spinal cord, the CPG located in the L1 and L2 spinal segments remains anatomically intact. Therefore, we propose the hypothesis that TNES, which has functions similar to those of EES, activates the CPG via sensory afferent axons to promote hindlimb movement [26,50]. In the present study, we observed intriguing results: under the synergistic effect of TNES, the rats’ BBB scores approached 12, and frequent coordinated forelimb–hindlimb movement emerged during weight-bearing locomotion. However, the structural basis underlying the synergistic effect between SCToid implantation and TNES-mediated modulation of sensorimotor neural circuits remains unclear. Further histological examination revealed that the combination of SCToid transplantation and TNES significantly promoted CST axon regeneration in the injury/graft site of the spinal cord and synaptic connectivity between regenerated axons and SCToid neurons. This facilitates the effective relay of brain-derived neural information to the sensorimotor neural circuits in the region caudal to the injury/graft site.

Several studies have showed that after the transplantation of homologous NSCs derived from caudalized (spinal cord) rather than rostralized (telencephalon) fates, the CST axons regenerated robustly to the injury/graft site and formed synapses with differentiated neurons to achieve structural integration [[51], [52], [53]]. However, the present study revealed that, in the E-SCToid group, numerous CST axons regenerated to the injury/graft site, exhibiting synaptic connectivity with SCToid neurons, indicating that the CST axons can achieve structural integration with the neurons differentiated from hippocampus-derived NSCs. The possible reason is that the SCToid neurons upregulate the expression of TrkC and integrin receptors, enabling enhanced synaptic formation potential and greater capacity to form synapses with CST axons—thereby supporting functional integration [44,54,55]. Therefore, it is not necessary, as some studies have suggested, to transplant the neurons differentiated from homologous NSCs to improve synaptic connectivity with regenerated brain-derived axons. Moreover, this study revealed that, under TNES stimulation, a greater number of BDA^+^ CST axons and CGRP^+^ sensory afferent axons established synaptic connectivity with SCToid neurons, indicating that SCToid neurons can effectively integrate neural information conveyed by both supraspinal (brain-derived) and sensory afferent pathways. Building upon this foundational evidence, the underlying mechanism by which SCToid neurons establish synaptic connectivity with sensorimotor neural circuits in the area caudal to the injury/graft site emerges as a critical research question, which will be addressed in the subsequent section.

### 4.3 Establishing synaptic connectivity between SCToid neurons and sensorimotor neural circuits

Although the repair strategy, in which neurons are transplanted at the injury site to relay neural information, has been proposed for many years and existing strategies have yielded regenerated CST axons at the injury/graft site, recovery of voluntary locomotor function in animals with complete SCI remains unsatisfactory [36,51]. The main reason is probably neurodegeneration and an imbalance in excitatory/inhibitory synaptic connectivity in the sensorimotor neural circuit caudal to the lesion [37,56,57].

We applied anterograde monosynaptic tracing to analyze the characteristics of SCToid integration with the sensorimotor neural circuit in the area caudal to the injury/graft site. The results revealed that excitatory neurons in the SCToid can establish monosynaptic connectivity with excitatory CPG interneurons (postsynaptic neurons), which are able to further innervate motor neurons after receiving excitatory neural information from SCToid neurons under the influence of TNES. Given that the E-SCToid group exhibited significant regeneration of brain-derived and sensory afferent axons that formed robust synaptic connectivity with SCToid neurons, these findings may partially explain why the E-SCToid group demonstrated larger CMEP amplitudes and shorter latencies compared to the SCToid group. We hypothesize that, by receiving and integrating brain-derived and proprioceptive sensory neural inputs, SCToid relay neurons may further activate excitatory CPG interneurons through monosynaptic connectivity to regulate voluntary locomotor function [38,39]. In addition, the proprioceptive sensory afferent axons derived from the tail nerve are able to innervate CPG interneurons via synaptic connectivity under TNES [33,48,58,59]. It is evident that the movement of the paralyzed hindlimbs, controlled by CPG interneurons, is synergistically regulated by SCToid relay neurons and TNES. Based on the time course of behavioral recovery in rats, it can be inferred that the regulation of coordinated movement between the forelimb–hindlimb movement by the neural stem cell-derived neuronal relayer and TNES reflects a process of synaptic connectivity reorganization over time [52,60].

### 4.4 TNES may stabilize the corticospinal tract-transplanted neuron-lumbar spinal cord sensorimotor neural network through neural regulation

Studies have showed that EES and pharmacological modulation significantly restore the excitatory/inhibitory balance of sensorimotor neural circuits in animals with incomplete SCI who had lost locomotor function and recovered movement ability in paralyzed limbs [19,37,61,62]. It is suggested that for transected SCI, on the basis of SCToid transplantation to reconstruct the neural pathway, the effective recovery of voluntary movement also requires synaptic plasticity underlying the excitatory/inhibitory balance in the sensorimotor neural circuit caudal to the spinal cord lesion site [63]. In the present study, after SCToid transplantation combined with TNES, the ratio of excitatory to inhibitory presynaptic terminals on the soma surface of CPG interneurons and motor neurons in the area caudal to the injury/graft site was close to that of normal rats. However, after resection of the SCToid graft, rats lost the ability to move their hindlimbs and exhibited a significant decrease in the number of excitatory presynaptic terminals on the soma surface of CPG interneurons and motor neurons, resulting in excitatory/inhibitory imbalance. Importantly, the above-mentioned change could not be reversed by subsequent TNES treatment. Together, these observations further confirm that the restoration of the excitatory/inhibitory balance in the sensorimotor neural circuit and recovery of voluntary locomotor function can be achieved through TNES on the basis of SCToid-led neural pathway reconstruction.

Our research demonstrates that SCToid transplantation, when combined with advanced EES or brain-computer-spinal cord interface technologies, can enhance the likelihood of sustained recovery of voluntary hindlimb locomotor function after severe SCI [20,37,61]. It is worthy of affirmation that TNES, with its non-invasive advantage, can be safely and effectively applied in the acute phase of SCI in rodents to prevent sensorimotor neural circuit functional silencing and skeletal muscle atrophy. In practice, this could represent a process of a transition from temporary to permanent walking capability [26]. In the future, transcutaneous lumbosacral nerve electrical stimulation or transcutaneous spinal cord electrical stimulation, which has similar functions to TNES, will be applied to activate the spinal sensorimotor neural circuits caudal to the lesion in patients with complete SCI, and to alleviate muscle atrophy in paralyzed legs, allowing patients time to wait for more advanced treatments.

## 5 Conclusions

The present study demonstrates that the SCToid can effectively replace injured spinal cord tissue and reconstruct neural pathways. Additionally, SCToid transplantation combined with TNES effectively activates the excitatory sensorimotor neural circuits below the injury/graft site, contributing to the restoration of excitatory/inhibitory balance. Specifically, the ratio of excitatory to inhibitory puncta on the soma surface was close to that of normal rats for both CPG interneurons and motor neurons (MNs) (Fig. 9h). The underlying mechanism by which spinal cord injury is repaired in this study may be as follows: after receiving and integrating brain-derived motor signals and sensory afferent inputs, SCToid neurons form synaptic connections with CPG-regulated sensorimotor neural circuits via axonal innervation, thereby restoring the excitatory/inhibitory balance of those circuits and enabling voluntary movement of the paralyzed hindlimbs under the synergistic effect of TNES. Our study provides a therapeutic strategy and theoretical foundation for the effective treatment of complete SCI, as well as for clinical intervention in sensorimotor neural circuit functional silencing or dysfunction.

## CRediT authorship contribution statement

**Bi-Qin Lai:** Conceptualization, Data curation, Formal analysis, Funding acquisition, Investigation, Project administration, Validation, Writing – original draft. **Chuang-Ran Wu:** Data curation, Formal analysis, Investigation, Methodology, Software, Validation, Visualization, Writing – original draft. **Shang-Bin Yang:** Data curation, Formal analysis, Investigation, Methodology, Project administration, Software, Writing – original draft. **Jing Xu:** Formal analysis, Investigation, Methodology, Software, Validation, Visualization. **Yue Yang:** Investigation, Methodology, Software, Validation, Visualization. **Rong-Jie Wu:** Investigation, Methodology, Validation, Visualization. **Hai-Yang Yu:** Investigation, Methodology, Validation, Visualization. **Zhen Chen:** Investigation, Methodology, Validation, Visualization. **Rui Liu:** Investigation, Methodology, Validation, Visualization. **Ying Ding:** Formal analysis, Funding acquisition, Resources, Validation. **Ge Li:** Formal analysis, Funding acquisition, Resources, Validation. **Xiang Zeng:** Formal analysis, Funding acquisition, Resources, Validation. **Yuan-Huan Ma:** Formal analysis, Funding acquisition, Resources, Validation. **Shan-Shan Ma:** Formal analysis, Funding acquisition, Resources, Validation. **Qiao-Ying Huang:** Formal analysis, Funding acquisition, Resources, Validation. **Ya-Qiong Wang:** Investigation, Methodology, Validation, Visualization. **Ling Zhang:** Formal analysis, Funding acquisition, Resources, Validation. **Zheng-Hong Chen:** Formal analysis, Funding acquisition, Resources, Validation. **Yi-Nan Guo:** Investigation, Methodology, Validation, Visualization. **Yuan-Feng Chen:** Formal analysis, Funding acquisition, Resources, Validation. **Jia-Feng Fang:** Conceptualization, Funding acquisition, Resources, Supervision, Validation, Writing – review & editing. **Qiu-Jian Zheng:** Conceptualization, Funding acquisition, Resources, Supervision, Validation, Writing – review & editing. **Yuan-Shan Zeng:** Conceptualization, Formal analysis, Funding acquisition, Project administration, Resources, Supervision, Validation, Writing – review & editing.

## Declaration of competing interest

The authors declare that they have no known competing financial interests or personal relationships that could have appeared to influence the work reported in this paper.

## Data Availability

Data will be made available on request.
