# Supplementary material for: Spinal cord tissueoid transplantation combined with tail nerve electrical stimulation promotes the voluntary movement of paralyzed hindlimbs in rats with transected spinal cord injury
Source: Mater Today Bio. 2026 Jun 29;39:103418. doi: 10.1016/j.mtbio.2026.103418 (PMC13351160; doi:10.1016/j.mtbio.2026.103418)
Supplement: Multimedia component 1 [file mmc1.docx]

**Supplementary material**

**Spinal cord tissueoid transplantation combined with tail nerve electrical stimulation promotes the voluntary movement of paralyzed hindlimbs in rats with transected spinal cord injury**

Bi-Qin Lai, Chuang-Ran Wu, Shang-Bin Yang, Jing Xu, Yue Yang, Rong-Jie Wu, Hai-Yang Yu, Zhen Chen, Rui Liu, Ying Ding, Ge Li, Xiang Zeng, Yuan-Huan Ma, Shan-Shan Ma, Qiao-Ying Huang, Ya-Qiong Wang, Ling Zhang, Zheng-Hong Chen, Yi-Nan Guo, Yuan-Feng Chen, Jia-Feng Fang, Qiu-Jian Zheng, Yuan-Shan Zeng

**Supplementary Figs. 1−14**


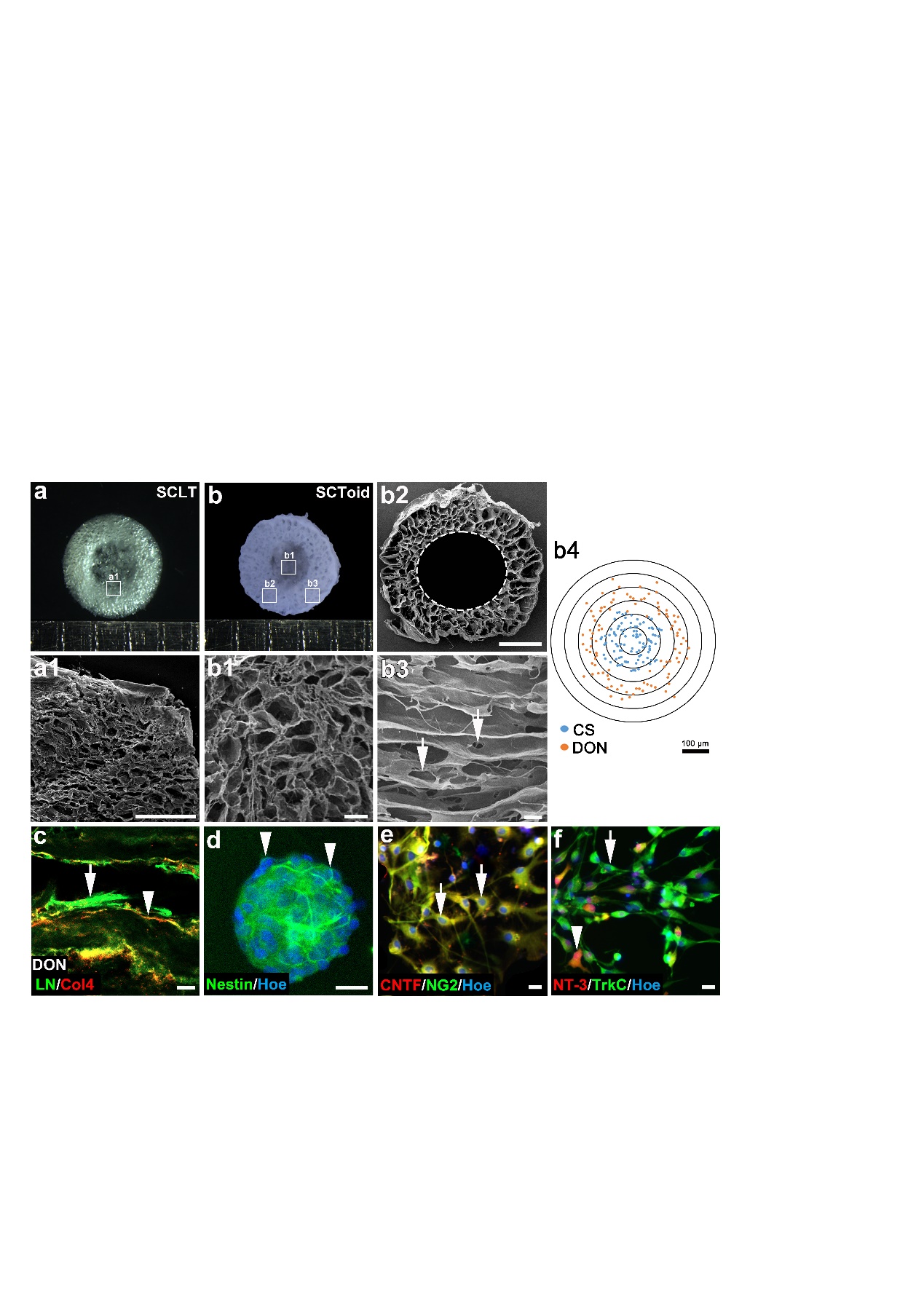


**Supplementary Fig. 1.** Tissue structure and cell identification of the SCToid. (a) SCLT consisted of circular CS scaffold and cylindrical CS scaffold under the light microscope, wherein irregular pores were observed under SEM (a1). (b−b4) SCToid was composed of circular DON scaffold and cylindrical CS scaffold (the three-dimensional structure showed in b1) under the light microscope and SEM. SEM images showed the cross-sectional (b2) and longitudinal-sectional (b3) views of the DON scaffold, revealing uniform longitudinal channels and small pores on the channel walls (indicated by arrows); the quantitative data on the pore size and dispersion of the CS and DON scaffolds were presented in (b4). (c) Abundant LN (arrow) and Col4 (arrowhead) were present on the surface of the DON scaffold channels. (d) Nestin^+^ NSCs (arrowheads) formed neurospheres. (e) NSCs expressed CNTF and NG2 after directed differentiation and AAV transfection (arrows). (f) NSCs expressed NT-3 (arrowhead) and TrkC (arrow) after AAV transfection. Cell nuclei were counterstained with Hoe. Scale bars = 1 mm (a, b, and b2), 500 μm (a1), 100 μm (b1, b3, and b4), 20 μm (c, d, and f), and 10 μm (e).


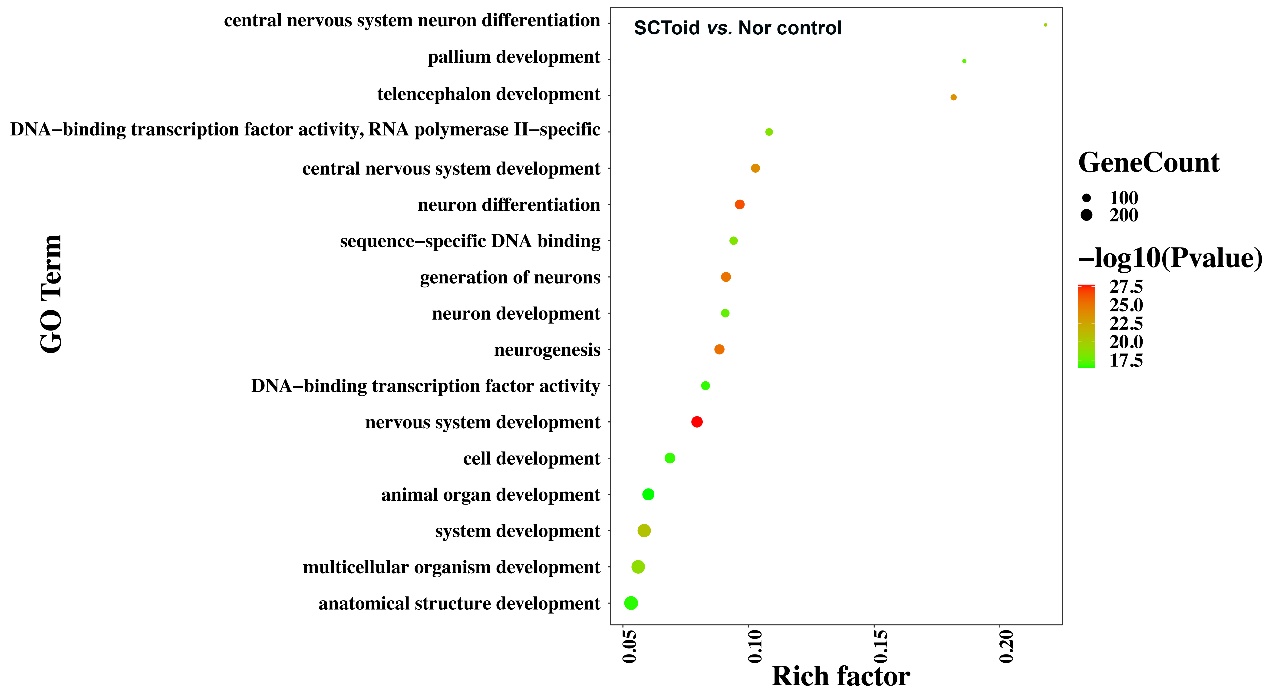


**Supplementary Fig. 2.** The GO enrichment bubble plot showed that the topGO terms in comparison of the SCToid group with the Nor control group were related mainly to nervous system development, neurogenesis and neuron differentiation.


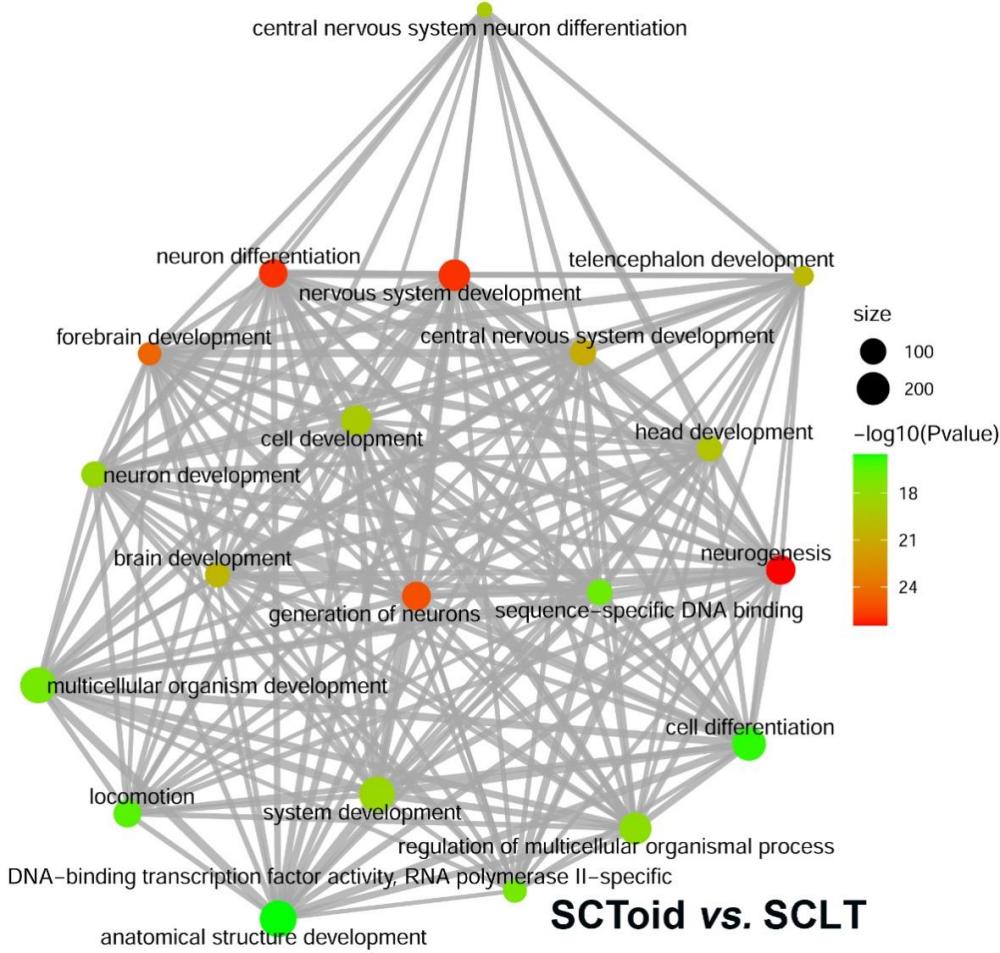


**Supplementary Fig. 3.** Network visualization of the top 20 GO categories and their overlap with upregulated differentially expressed genes between the SCToid and SCLT groups, with node sizes representing the total number of candidate genes within the GO category and colour indicating the -log10 (*P* value).


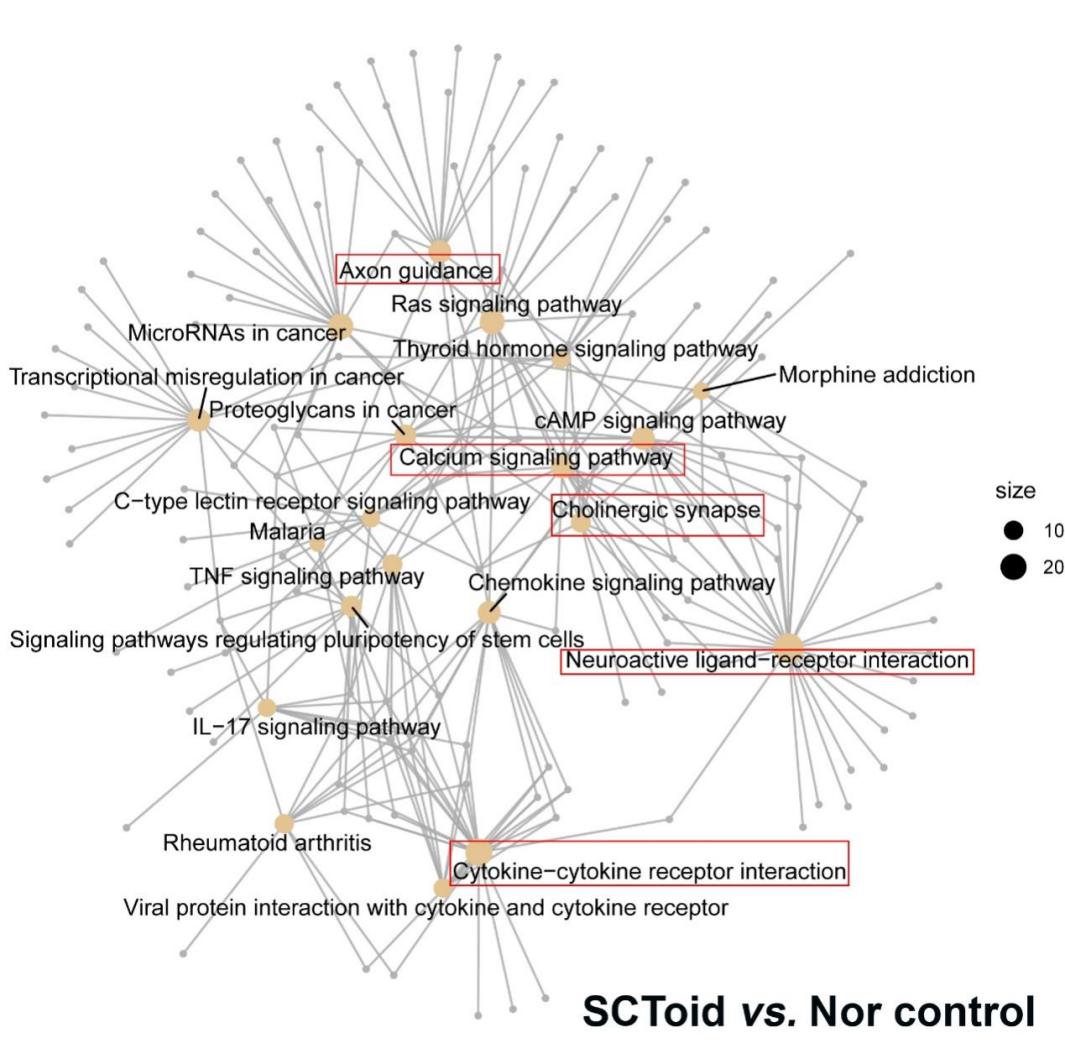


**Supplementary Fig. 4.** Network diagram showing the association of enriched genes in comparison of the SCToid group with the Nor control group. The node size of the functional set represents the total number of candidate genes according to gene ontology.


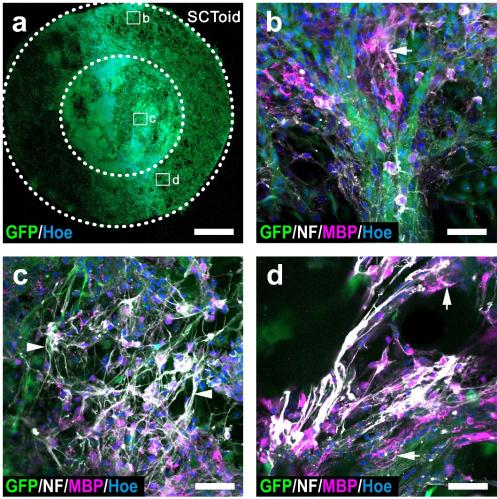


**Supplementary Fig. 5.** (a−d) The neurons in the GMLT of the SCToid (a) extended many NF^+^ neurites (arrowheads, c) into the WMLT. Some neurites (arrows) were surrounded by MBP^+^ myelin sheaths (b, d). Scale bars = 500 μm (a) and 20 μm (b−d).


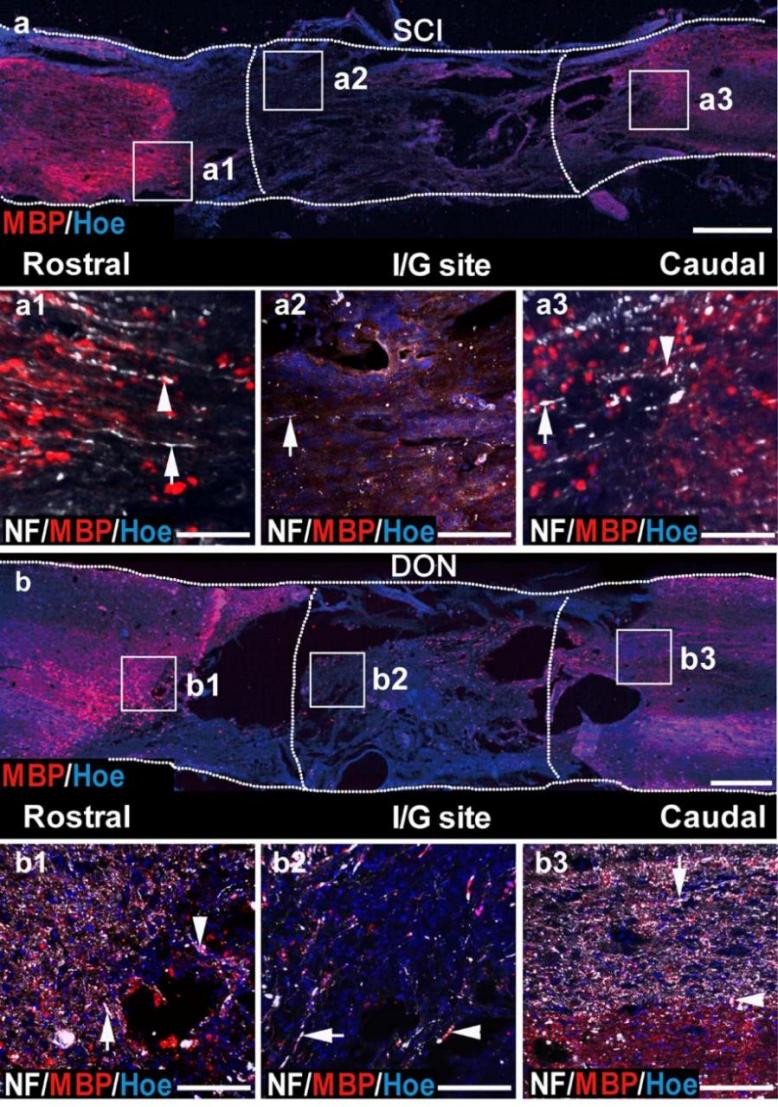


**Supplementary Fig. 6.** Nerve regeneration and myelination in the SCI and DON groups. (a, b) Low-magnification images of sagittal spinal cord sections showed the expression of NF and MBP in the SCI (a) and DON (b) groups. (a1−a3, b1−b3) Higher magnification image of the boxed areas in (a, b) showed the NF^+^ axons (arrows) and MBP^+^ myelin sheaths (arrowheads) in the areas rostral (a1, b1) and caudal (a2, b2) to/in the injury/graft sites (a3, b3). Scale bars = 500 μm (a and b) and 50 μm (a1−a3 and b1−b3).


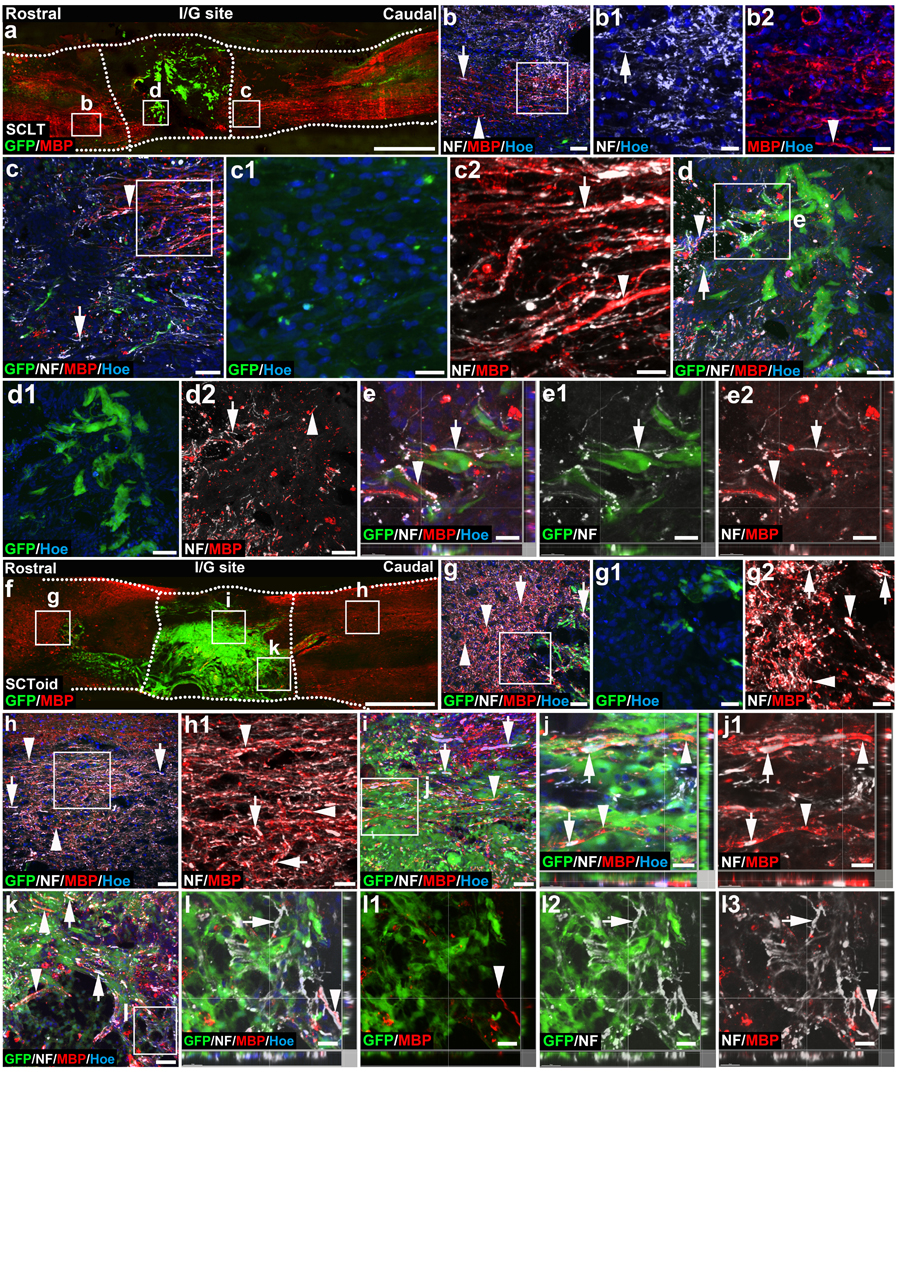


**Supplementary Fig. 7.** Nerve regeneration and myelination in the SCLT and SCToid group. (a) Low-magnification image of a sagittal spinal cord section showed GFP^+^ transplanted cells and the expression of MBP in the SCLT group. (b) Higher magnification image of the boxed area in (a) showed the NF^+^ axons (arrows) and MBP^+^ myelin sheaths (arrowheads) in the areas rostral (b, b1, b2) and caudal (c, c1, c2) to the injury/graft site and the injury/graft site (d, d1, d2). (e, e1, e2) Higher magnification image of the boxed area in (d) showed GFP^+^ cell contacting with NF^+^ axon (arrow), which was not surrounded by MBP^+^ myelin sheath. (f) Low-magnification image of a sagittal spinal cord section showed GFP^+^ transplanted cells and the expression of MBP in the SCToid group. Higher magnification image of the boxed area in (g) showed NF^+^ axons (arrows) and MBP^+^ myelin sheaths (arrowheads) in the areas rostral (g1, g2) and caudal (h, h1) to the injury/graft site and in the injury/graft site (i, k). (i, j, j1) Higher magnification image of the boxed area in (f) showed that regenerated NF^+^ axons (arrows) were surrounded by MBP^+^ myelin sheaths (arrowheads) on the dorsal side of the injury/graft site. (k) Higher magnification image of the boxed area in (f) revealed abundant regeneration of NF^+^ axons (arrows), accompanied by the presence of MBP^+^ myelin sheaths (arrowheads) on the ventral side of the injury/graft site (l, l1−l3). Scale bars = 1 mm (a and f), 50 μm (b, c, d−d2, g, h, i, and k), and 20 μm (b1, b2, c1, c2, e−e2, g1, g2, h1, j, j1,1, and l1−l3).


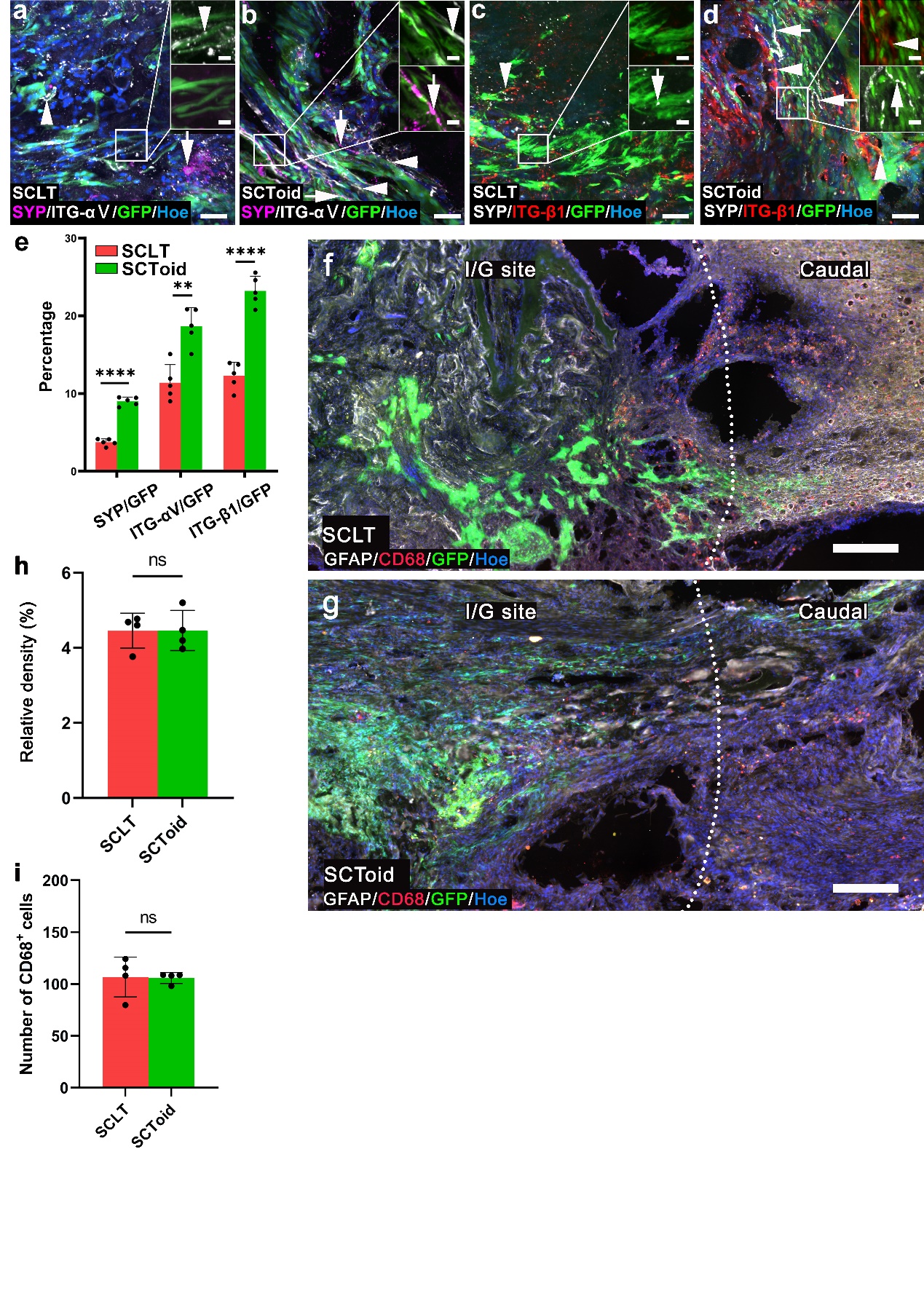


**Supplementary Fig. 8.** (a, b) Colocalization of GFP^+^ cells with SYP (arrows) and ITG-αv (arrowheads) was observed in the injury/graft site in the SCLT (a) and SCToid (b) groups. (c, d) Colocalization of GFP^+^ cells with SYP (arrows) and ITG-β1 (arrowheads) was observed in the injury/graft site in the SCLT (c) and SCToid (d) groups. (e) Relative density analysis revealed that the area of SYP^+^, ITG-αv^+^ and ITG-β1^+^ fluorescence within the area occupied by GFP^+^ cells in the injury/graft site was significantly higher in the SCToid group (*n* = 5; Student’s t-tests were applied for comparisons between two groups, ***P* < 0.01, *****P* < 0.0001). (f–i) Distribution of GFAP^+^ astrocytes (h) and CD68^+^ cells (i) in SCLT (f) and SCToid (g), with corresponding quantification at 8 weeks post-transplantation (*n* = 4; Student’s t-tests were applied for comparisons between two groups; ns: not significant). Cell nuclei were counterstained with Hoe. Scale bars = 50 μm (a−d), 10 μm (box area in a−d), and 200 μm (f and g).


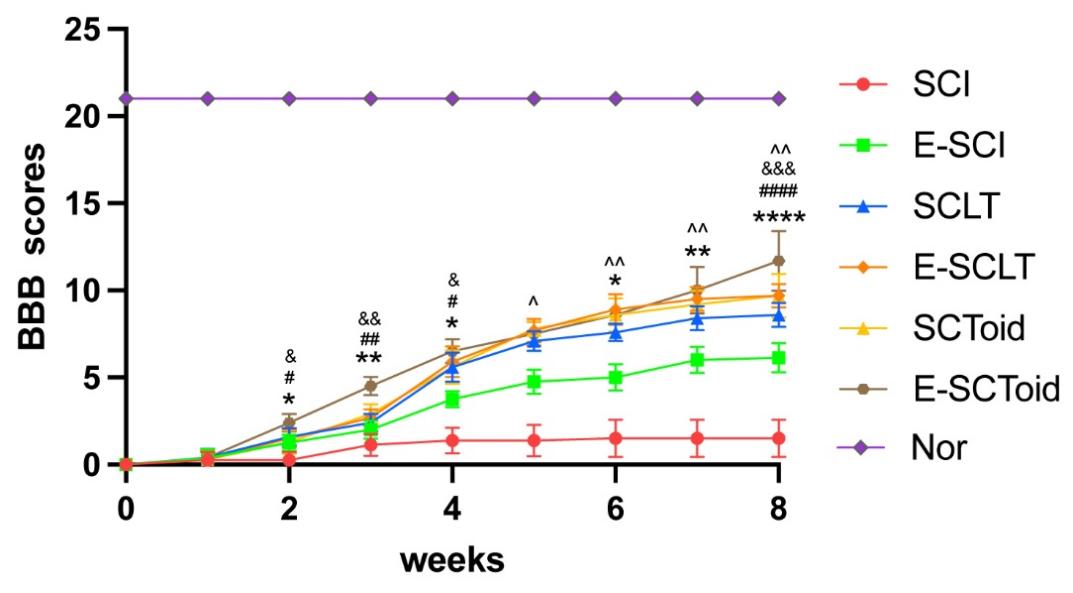


**Supplementary Fig. 9.** Behavioral analysis was performed in rats following SCI (*n* = 8), SCLT (*n* = 8) and SCToid (*n* = 10) transplantation combined with TNES treatment. BBB scores were assessed for the E-SCI (*n* = 8), E-SCLT (*n* = 8) and E-SCToid (*n* = 10) groups from 1 to 8 weeks post-treatment (one-way ANOVA with LSD-t post-hoc test at each time point, * indicates statistical significance when E-SCToid group is compared with the E-SCLTgroup; ^#^ indicates statistical significance when E-SCToid group is compared with the SCLT group, & indicates statistical significance when E-SCToid group is compared with the SCToid group, ^ indicates statistical significance when E-SCLT group is compared with the SCLT group; **P* < 0.05, ***P* < 0.01, *****P* < 0.0001, ^#^*P* < 0.05, ^##^*P* < 0.01, ^####^*P* < 0.0001, ^&^*P* < 0.05, ^&&^*P* < 0.01, ^&&&^*P* < 0.001, ^*P* < 0.05, ^^^^*P* < 0.01).


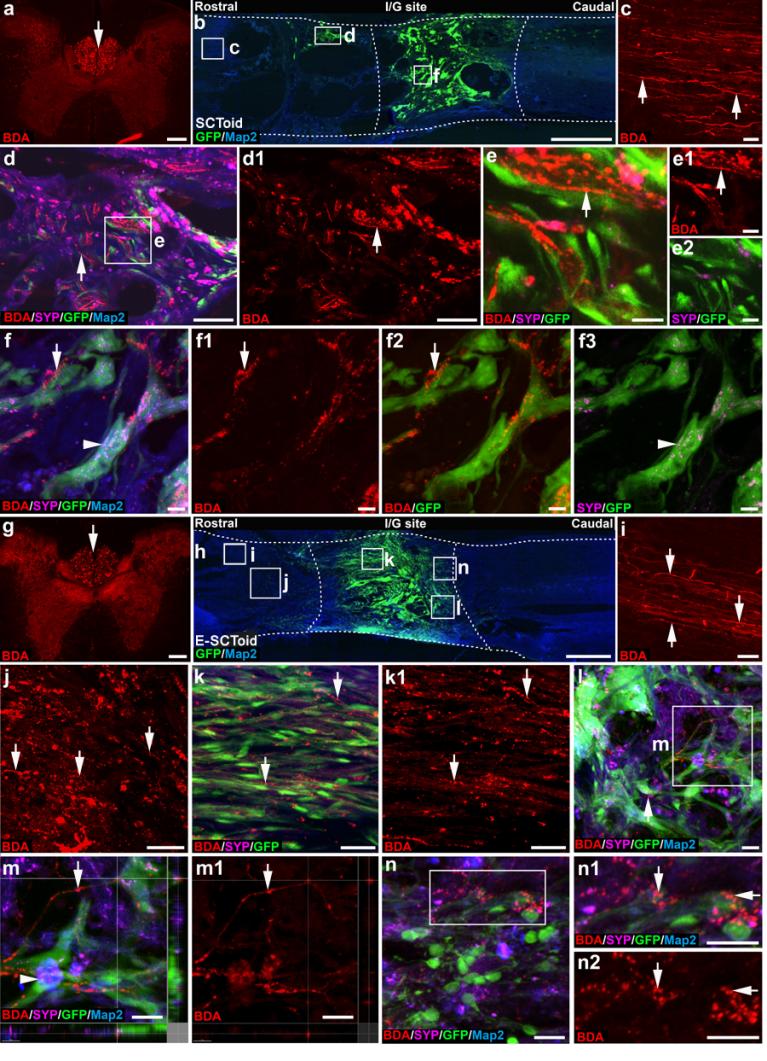


**Supplementary Fig. 10.** BDA was used to trace the CST axons distributed in the injured spinal cord in the SCToid and the E-SCToid groups. (a) Cross-section of the cervical spinal cord in the SCToid group showed the distribution of BDA^+^ CST axons (arrows). (b) Sagittal-section of the spinal cord containing the injury/graft site in the SCToid group. (c) A higher magnification image of the boxed area in (b) showed the distribution of BDA^+^ CST axons (arrows) in the area rostral to the injury/graft site of spinal cord. (d) Another higher magnification image of the boxed area in (b) displayed the distribution of BDA^+^ CST axons (arrow, d1) in the rostral of the injury/graft site, where GFP^+^ cells were present. (e) Higher magnification image of the boxed area in (d) highlighted the mutual contaction between BDA^+^/SYP^+^ CST axons (arrow) and GFP^+^ cells (e1, e2). (f) Higher magnification image of the boxed area in (b) showed the mutual contaction between BDA^+^ CST axons (arrows, f1, f2) and GFP^+^/MAP2^+^ cells (arrowhead, f) in the central of the injury/graft site, as well as the SYP markers (arrowhead, f3) detected at the sites in contact with GFP^+^/MAP2^+^ cells (arrowhead, f). (g) Cross-section of the cervical spinal cord showed the distribution of BDA^+^ CST axons (arrow) in the E-SCToid group. (h) Sagittal-section of the spinal cord containing the injury/graft site in the E-SCToid group. (i, j) Higher magnification image of the boxed area in (h) showed the distribution of BDA^+^ CST axons (arrows) in the area rostral to the injury/graft site of spinal cord. (k) Higher magnification image of the boxed area in (h) displayed the distribution of BDA^+^ CST axons (arrows, k1) in the injury/graft site, where GFP^+^ cells were present. (l) Higher magnification image of the boxed area in (h) highlighted the mutual contaction between GFP^+^/MAP2^+^ cells (arrowhead) and BDA^+^ CST axons (arrows, m, m1) in the caudal of the injury/graft site, as well as the SYP markers detected at the contact sites (arrowhead) with GFP^+^/MAP2^+^ cells (arrowhead, m). (n) Higher magnification image of the boxed area in (h) showed the mutual contaction between GFP^+^ cells and BDA^+^ CST axons (arrows, n1, n2) in the caudal of the injury/graft site. Scale bars = 1 mm (b and h), 500 μm (a and g), 50 μm (c, d, d1, and i−l), 20 μm (f−f3), and 10 μm (e−e2, m, m1, and n−n2).


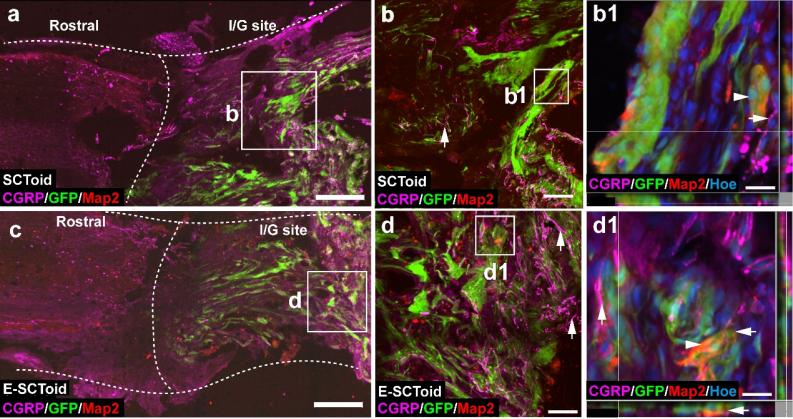


**Supplementary Fig. 11.** (a−b1) Showing that regenerated CGRP^+^ axons (arrow, b) closely contacted the Map2^+^ transplanted neurons (arrowhead, b1) in the injury/graft site in the SCToid group, and the enlarged image exhibits CGRP^+^ axons (arrows, b1) at the contact site. (c−d1) In the E-SCToid group, CGRP^+^ axons (arrows) regenerated significantly into the injury/graft site (d), and the enlarged image exhibited that CGRP^+^ axons (arrows, d1) closely contacted the Map2^+^ transplanted neurons (arrowhead, d1). Scale bars = 500 μm (a and c), 100 μm (b and d), and 20 μm (b1 and d1).


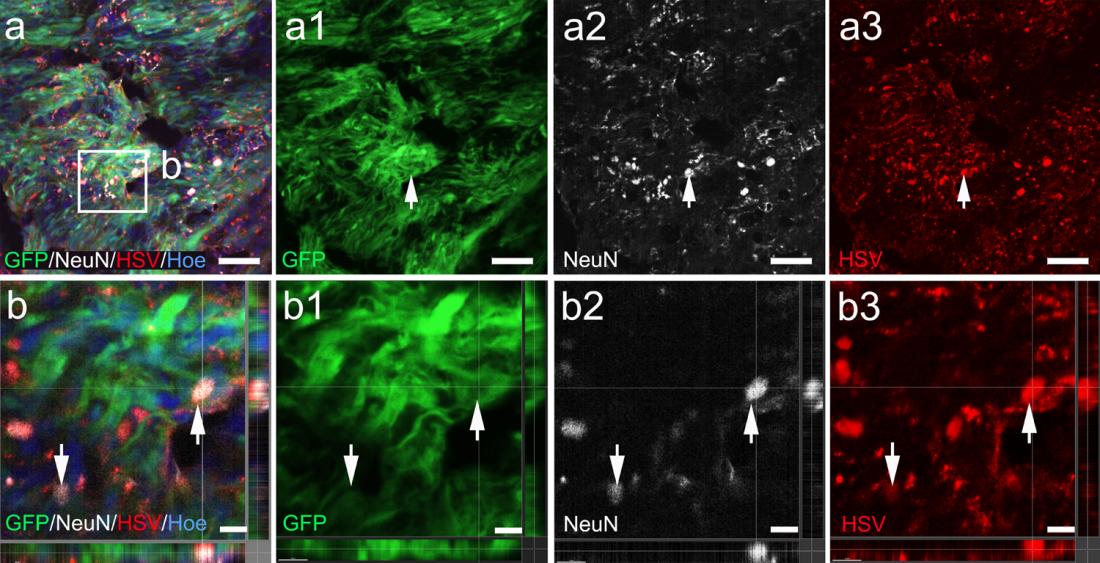


**Supplementary Fig. 12.** Displaying the expression of NeuN in GFP^+^ neurons in the SCToid group. (a) In the injury/graft site of spinal cord in the E-SCToid group, the GFP^+^ cells (arrow, a1) were observed to express NeuN (arrow, a2) and labeled by HSV (arrow, a3). (b) A higher magnification image of the boxed area in (a) showed that transplanted GFP^+^ neurons (arrows, b1) expressed NeuN (arrows, b2) and were labeled by HSV (arrows, b3). Scale bars = 50 μm (a−a3) and 10 μm (b−b3).


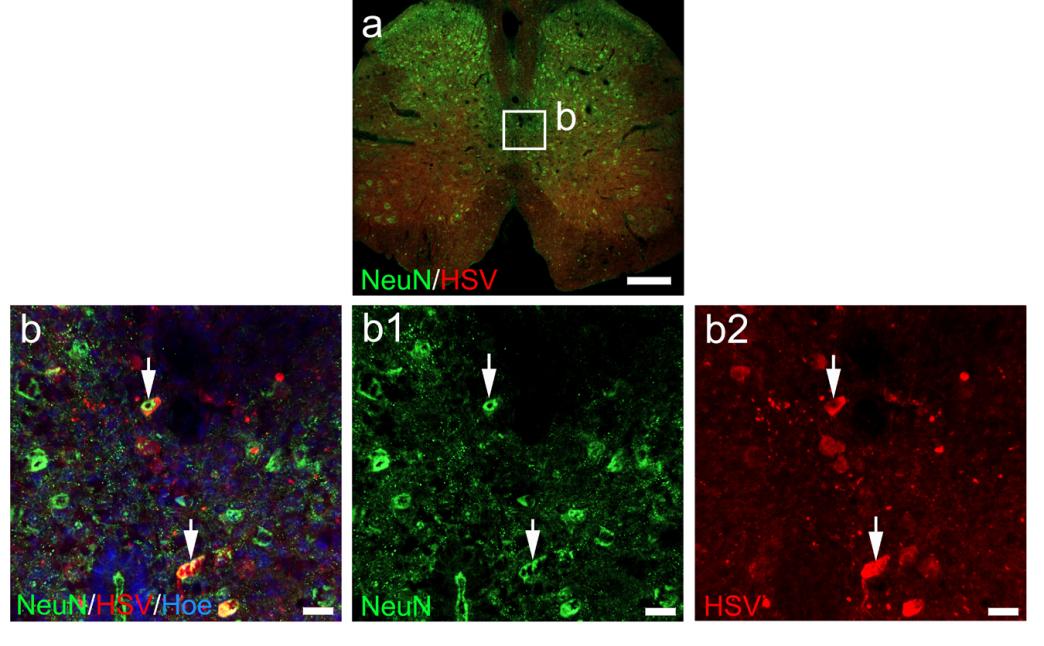


**Supplementary Fig. 13.** The expression of NeuN was detected in neurons infected with HSV in the lumbar spinal cord in the SCToid group. (a) Cross-section of the L3 spinal cord segment. (b) A higher magnification image of the boxed area in (a) showed expression of NeuN (arrows, b1) in neurons infected with HSV (arrows, b2). Scale bars = 300 μm (a) and 20 μm (b−b2).


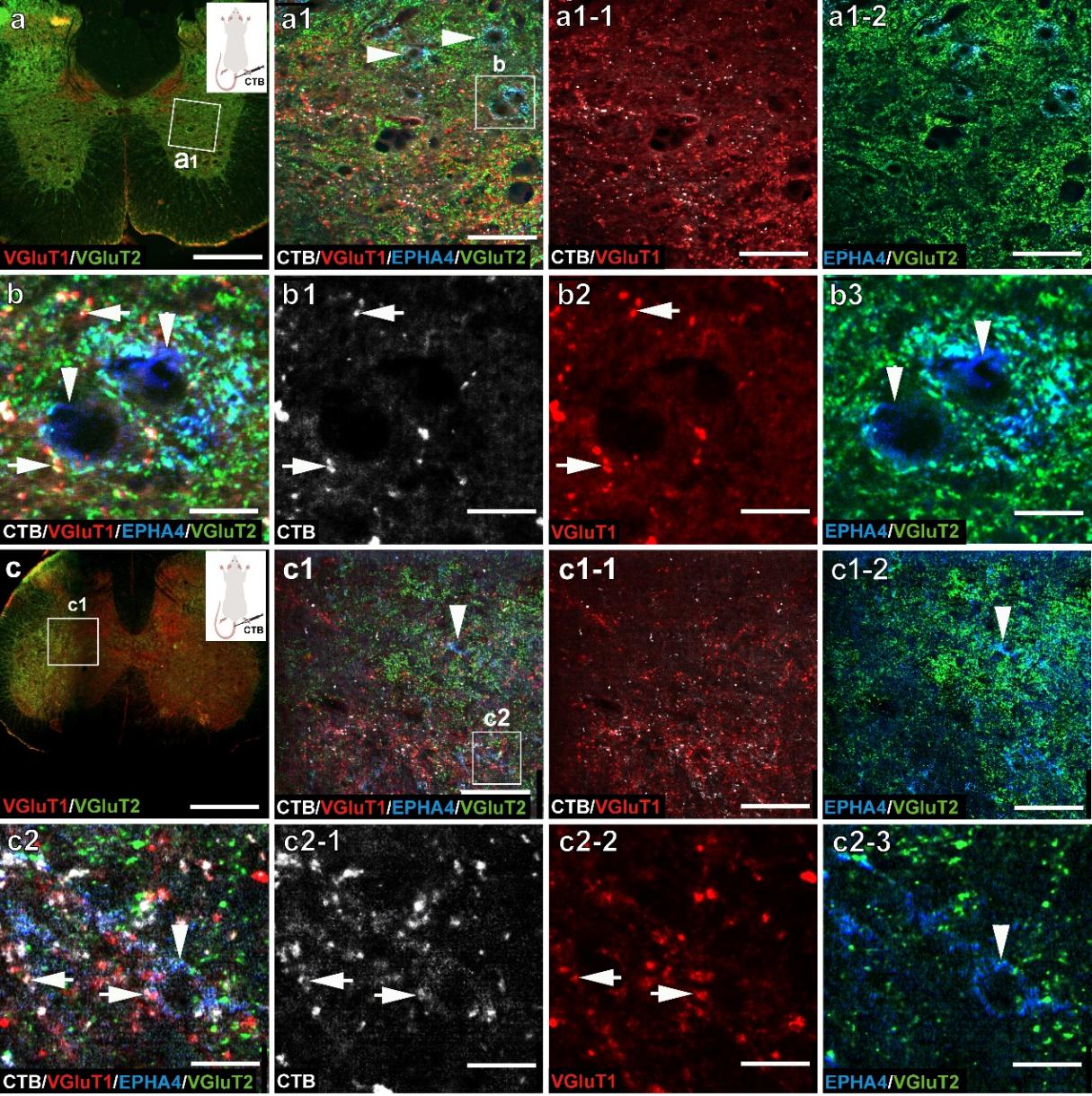


**Supplementary Fig. 14.** (a) The image exhibited the cross-section of L2 spinal cord segment after tail nerve CTB tracing in the E-SCToid group. (a1) Higher magnification image of the boxed area in (a) showed that EPHA4^+^/VGluT2^+^ neurons (arrowheads) colocalized with CTB^+^/VGluT1^+^ axons. Further magnification image revealed the distribution of CTB^+^/VGluT1^+^ axon terminals (arrows, b−b2) on the soma surface of EPHA4^+^/VGluT2^+^ neurons (arrowheads, b, b3). (c) The image exhibited the cross-section of L4 spinal cord segment after tail nerve CTB tracing in the E-SCToid group. (c1) Higher magnification image of the boxed area in (c) showed EPHA4^+^/VGluT2^+^ neurons (arrowheads, c1, c1-2) and CTB^+^/VGluT1^+^ axons. Further magnification image revealed the distribution of CTB^+^/VGluT1^+^ axon terminals (arrows, c2−c2-2) on the soma surface of EPHA4^+^/VGluT2^+^ neurons (arrowheads, c2, c2-3). Scale bars = 500 μm (a and c), 50 μm (a1−a1-2 and c1−c1-2), and 10 μm (b−b3 and c2−c2-3).

**Supplementary Table 1.** Treatment group compositions

| Groups | Surgery/  Transplantation | Therapeutic Strategy | Behavior Test | Neural Tracing |
| --- | --- | --- | --- | --- |
| Normal | none | none | Open-field locomotor test;  Grid climbing test | none |
| SCI | T10 complete SCI | none | Open-field locomotor test;  Grid climbing test | none |
| E-SCI | T10 complete SCI | Tail nerve electrical stimulation | Open-field locomotor test;  Grid climbing test | none |
| DON | T10 complete SCI+DON transplantation | none | Open-field locomotor test;  Grid climbing test | none |
| SCLT | T10 complete SCI+SCLT transplantation | none | Open-field locomotor test;  Grid climbing test | none |
| E-SCLT | T10 complete SCI+SCLT transplantation | Tail nerve electrical stimulation | Open-field locomotor test;  Grid climbing test | none |
| SCToid | T10 complete SCI+SCToid transplantation | none | Open-field locomotor test;  Grid climbing test | BDA (biotin dextran amine) tracing |
| E-SCToid | T10 complete SCI+SCToid transplantation | Tail nerve electrical stimulation | Open-field locomotor test;  Grid climbing test | BDA-tracing; Anterograde transmonosynaptic tracing;  CTB (cholera toxin B) tracing |
| 1. SCToid-   cut | T10 complete SCI+SCToid transplantation | Tail nerve electrical stimulatio + SCToid graft cuted at 8W | Open-field locomotor test;  Grid climbing test | none |

| **Supplementary Table 2.** Primary and secondary antibodies | | | | |
| --- | --- | --- | --- | --- |
| Antibodies | Species | Type | Dilution | Source (Catalog) |
| Neurofilament 200 (NF) | Chicken | Polyclonal IgG | 1:500 | Abcam, London, UK (ab4680) |
| NeuN | Rabbit | Polyclonal IgG | 1:200 | Cell signaling Technology, Massachusetts, USA (12943S) |
| Synaptophysin (SYP) | Mouse | Monoclonal IgG | 1:200 | Sigma, St. Louis, USA (S5768) |
| Synaptophysin-647 (SYP-647) | Rabbit | Monoclonal IgG | 1:1000 | Abcam, London, UK (ab196166) |
| Microtubule-associated protein 2 (Map2) | Mouse | Monoclonal IgG | 1:1000 | Sigma, St. Louis, USA (M4403) |
| Glial fibrillary acidic protein (GFAP) | Rabbit | Polyclonal IgG | 1:2000 | Abcam, London, UK (ab7260) |
| Nestin | Mouse | Monoclonal IgG | 1:500 | Abcam, London, UK (ab6142) |
| Laminin (LN) | Rabbit | Polyclonal IgG | 1:1000 | Sigma, St. Louis, USA (L9393) |
| Col4 | Mouse | Monoclonal IgG | 1:500 | Santa Cruz, Texas, USA (sc-517572) |
| Ciliary neurotrophic factor (CNTF) | Rabbit | Polyclonal IgG | 1:500 | Abcam, London, UK (ab46172) |
| calcitonin gene related peptide (CGRP) | Mouse | Monoclonal IgG | 1:1000 | Abcam, London, UK (ab81887) |
| Myelin basic protein (MBP) | Rabbit | Polyclonal IgG | 1:400 | Merck Millipore, Billerica, USA (AB980) |
| TrkC | Goat | Polyclonal IgG | 1:300 | Sigma, St. Louis, USA (T2450) |
| Neurotrophin-3 (NT-3) | Rabbit | Polyclonal IgG | 1:300 | Sigma, St. Louis, USA (SAB1300907) |
| ITG-β1 | Mouse | Monoclonal IgG | 1:100 | Santa Cruz, Texas, USA (sc-374429) |
| ITG-αv | Rabbit | Monoclonal IgG | 1:200 | Abcam, London, UK (ab222222) |
| Postsynaptic density protein 95 (PSD95) | Rabbit | Polyclonal IgG | 1:800 | Abcam, London, UK (ab18258) |
| Erythropoietin-producing hepatocellular receptor A4 (EPHA4) | Mouse | Monoclonal IgG | 1:100 | Santa Cruz, Texas, USA (sc-135897) |
| Vesicular glutamate transporter 1 (VGluT1) | Mouse | Monoclonal IgG | 1:2500 | Abcam, London, UK (ab242204) |
| Vesicular glutamate transporter 2 (VGluT2) | Rabbit | Monoclonal IgG | 1:5000 | Abcam, London, UK (ab216463) |
| Choline acetyltransferase (ChAT) | Rabbit | Polyclonal IgG | 1:800 | Merck Millipore, Billerica, USA (AB2219) |
| Vesicular GABA transporter (VGAT) | Mouse | Monoclonal IgG | 1:500 | SYNAPTIC SYSTEMS (131011) |
| GAPDH | Mouse | Monoclonal IgG | 1:10000 | Abcam, London, UK (ab8245) |
| Oligodendrocyte transcription factor 2 (Olig2) | Rabbit | Monoclonal IgG | 1:5000 | Abcam, London, UK (ab109186) |
| Streptavidin  Conjugate555 |  |  | 1:300 | Invitrogen, USA (S32355) |
| Alexa fluor 488 goat anti-chicken secondary antibody | Goat | Polyclonal IgG | 1:800 | Abcam, London, UK (ab150169) |
| Alexa fluor 488 goat anti-rabbit secondary antibody | Goat | Polyclonal IgG | 1:800 | Abcam, London, UK (ab150077) |
| Alexa fluor 488 goat anti-mouse secondary antibody | Goat | Polyclonal IgG | 1:800 | Abcam, London, UK (ab150113) |
| Alexa fluor 555 goat anti-mouse secondary antibody | Goat | Polyclonal IgG | 1:800 | Abcam, London, UK (ab150114) |
| Alexa fluor 555 goat anti-rabbit secondary antibody | Goat | Polyclonal IgG | 1:800 | Abcam, London, UK (ab150169) |
| Alexa fluor 647 goat anti-mouse secondary antibody | Goat | Polyclonal IgG | 1:800 | Abcam, London, UK (ab150115) |
| DyLigh 405 goat anti-rabbit secondary antibody | Goat | Polyclonal IgG | 1:500 | Jackson ImmunoResearch, West  Grove, USA (111-475-003) |
| Alexa fluor 647 goat anti- rabbit secondary antibody | Goat | Polyclonal IgG | 1:800 | Abcam, London, UK (ab150079) |
| [Goat anti-mouse HRP](http://www.abcam.com/goat-mouse-igg-hl-hrp-ab6789.html) | Goat | Polyclonal IgG | 1:2000 | Abcam, London, UK (ab6789) |
| [Goat anti-rabbit HRP](http://www.abcam.com/goat-mouse-igg-hl-hrp-ab6789.html) | Goat | Polyclonal IgG | 1:2000 | Abcam, London, UK (ab6721) |
